# MAGUK p55 subfamily member 7 attenuates allergic airway inflammation by modulating lung dendritic cells functions

**DOI:** 10.1038/s41598-026-40491-w

**Published:** 2026-02-28

**Authors:** Yingli Men, Yongfeng Chen, Yi Shao, Hui Wang, Guoxue Wu, Zhenzhen Yang, Zhaorui Wang, Dong Liu, Ping Wang, Yahui Hu, Yang Zheng, Xiaoyan Xu, Miling Yang, Huang Jiang, Yinsen Song, Cong Ding

**Affiliations:** 1 Translational Medicine Research Center, The Fifth Clinical Medical College of Henan, University of Chinese Medicine (Zhengzhou People’s Hospital), Zhengzhou, Henan China; 2 Information Department, Henan Cancer Hospital, Zhengzhou, Henan China; 3 Dermatology Department, Henan Provincial People’s Hospital, Zhengzhou, Henan China; 4 Respiratory and immune Diseases Laboratory, Children’s Hospital Affiliated to Zhengzhou University, 33 Longhu Waihuan East Road, Zhengzhou, 450018 Henan P.R. China; 5 Pharmacy Department, The Fifth Clinical Medical College of Henan University of Chinese Medicine (Zhengzhou People’s Hospital), Zhengzhou, Henan China; 6 Pathology Department, The Fifth Clinical Medical College of Henan University of Chinese Medicine (Zhengzhou People’s Hospital), Zhengzhou, Henan China; 7 Laboratory Department, The Fifth Clinical Medical College of Henan University of Chinese Medicine (Zhengzhou People’s Hospital), Zhengzhou, Henan China

**Keywords:** MPP7, DCs, Allergic response, Diseases, Immunology

## Abstract

Asthma remains the most prevalent chronic respiratory condition affecting pediatric populations, with no curative therapy currently available. Dendritic cells (DCs) are pivotal in initiating Th2 polarization during asthmatic responses, though molecular mechanisms governing DC activation remain incompletely elucidated. As a constituent of the membrane palmitoylated protein family, membrane-associated guanylate kinase (MAGUK) p55 subfamily member 7 (MPP7) has potential therapeutic implications for respiratory disorders, yet its specific involvement in asthma pathogenesis requires further investigation. This study aimed to elucidate MPP7’s protective effects against allergic airway inflammation by analyzing its impact on Th2 immune responses and DC functionality. Clinical data revealed notably reduced MPP7 levels in asthmatic patients’ peripheral blood samples. Experimental models using house-dust-mite (HDM)-exposed mice demonstrated that MPP7 deficiency intensified airway inflammatory responses, with pathological manifestations including amplified leukocyte infiltration, heightened Th2 cytokine production, and elevated serum levels of HDM-specific IgE. Notably, MPP7 caused a marked reduction in pulmonary CD11b^+^CD103^−^ DCs populations while inhibiting DC activation via interference with NF-κB pathway signaling. These results collectively suggest that MPP7 exerts its anti-inflammatory effects through dual mechanisms involving both cellular subset modulation and intracellular signaling pathway inhibition in DCs, positioning it as a promising candidate for managing allergic respiratory disorders and associated immune-mediated conditions.

**Supplementary Information:**

The online version contains supplementary material available at 10.1038/s41598-026-40491-w.

##  Introduction

Asthma represents one of the most prominent chronic respiratory conditions contributing to disease burden across pediatric and adult demographics globally. According to the World Health Organization (WHO) statistics from 2016, the global asthma prevalence reached 339 million cases, with mortality data from 2019 recording 461,000 asthma-associated fatalities^1^. Research published in The Lancet (2019) revealed that 4.20% of Chinese adults aged 20 years or older exhibited asthma symptoms, equating to approximately 45.7 million affected individuals^2^. Parallel epidemiological data demonstrated a 3.02% asthma occurrence rate among Chinese children under 14 years, affecting nearly 15 million pediatric cases^3^. While current therapeutic approaches including inhaled corticosteroids and supplementary treatments remain effective in symptom management, their capacity to modify the disease’s relapsing-remitting trajectory and long-term progression appears constrained^4^. This therapeutic limitation underscores the critical need for advancing the understanding of distinct asthma subtypes and their molecular pathogenesis, which could facilitate the creation of targeted treatment protocols.

In the pathogenesis of asthma, lung DCs are now regarded as the core regulatory factors triggering the T-helper 2 (Th2) immune response^5^. When exogenous antigens enter the body through the respiratory tract, the DCs in the tracheal lumen will capture and process them. These antigen-activated DCs then migrate to regional lymph nodes and convey the processed antigen information to immature CD4^+^ T lymphocytes through antigen presentation^6^. During this process, DCs express co-stimulatory molecules (such as CD86 and CD40) and secrete specific cytokines (including IL-6 and TNF-α). This drives the differentiation of various T cell subsets such as Th1, Th2, and Th17. Among them, Th2 cells significantly exacerbate the inflammatory cascade response of allergic asthma by secreting cytokines such as IL-4, IL-5, and IL-13. Current research has confirmed that among the molecular systems related to the regulation of DCs function, only meteorin-β (METRN-β) contactin-1 (CNTN1) and membrane-associated ring finger structural protein 1 (MARCH1) have been clearly involved in the asthma process^7–9^. Specifically, METRN-β exerts disease remission by antagonizing the Th2 response mediated by DCs, while CNTN1 and MARCH1 exacerbate pathological damage by enhancing the Th2 differentiation process mediated by DCs. Despite advances in understanding the underlying mechanisms, these molecules have not yet been successfully applied to clinical applications as diagnostic biomarkers. Consequently, there remains a critical need to discover novel molecular profiles originating from dendritic cells, which represents an essential requirement for improving diagnostic approaches and developing targeted treatment strategies for asthma. The current gap between mechanistic discoveries and clinical implementation highlights the importance of identifying reliable DC-derived biomarker candidates to bridge this translational divide in respiratory medicine.

In parallel, epithelial barrier integrity and polarity are increasingly recognized as upstream determinants of allergen responsiveness^10^. Proteins that organize tight junctions and polarity complexes do not merely maintain structure; they can scaffold signaling platforms that influence how tissues and immune cells respond to environmental cues. The membrane-associated guanylate kinase (MAGUK) family member MPP7 functions as a scaffolding protein by coordinating four modular domains—L27, PDZ, SH3, and GUK—to organize multiprotein complexes that regulate cellular proliferation, adhesion, junctional dynamics, and apical-basal polarity^11^. Epithelial development studies demonstrate that MPP7 collaborates with the polarity regulator discs large homolog 1 (Dlg1) through ternary complex formation, which stabilizes tight junction architecture and promotes apical membrane specialization^12^. Notably, bioinformatics analyses in cancer have found correlations between MPP7 expression and immune cell infiltration, suggesting that MPP7 might influence inflammatory or immune processes^13–15^. These insights collectively highlight MPP7’s multifunctional role in maintaining cellular homeostasis, though its involvement in asthma-related immune mechanisms remains unexplored. This investigation utilized MPP7-deficient (MPP7^−/−^) mice combined with a house dust mite (HDM)-driven murine asthma model and ex vivo cultured bone marrow derived dendritic cells (BMDCs) to investigate the function of MPP7 in alleviating Th2-mediated airway inflammatory responses and influencing the development, functional characteristics, and antigen-presenting capabilities of both lung-resident DCs and BMDCs.

##  Methods

### Assessment of MPP7 levels in human peripheral blood samples

All procedures were performed following relevant National Institutes of Health guidelines and approved by the Research Ethics Committee at Henan University of Chinese Medicine’s Zhengzhou People’s Hospital (Ethics No. 2025-KY-003801). The study enrolled ten asthma patients and ten healthy controls. Diagnostic confirmation for asthma participants adhered strictly to the Global Initiative for Asthma (GINA) criteria. Prior to participation, written informed consent was obtained from all subjects during the recruitment phase. Blood samples collected in heparinized tubes underwent immediate centrifugation (1000 × g, 10 minutes, 4 °C) to isolate cellular components. TRIzol reagent (Invitrogen) facilitated RNA extraction from harvested cells, followed by cDNA synthesis using Thermo Fisher Scientific’s reverse-transcription kit. Serial dilutions of synthesized cDNA were prepared for subsequent quantitative PCR (qPCR) analysis. Primer sequences used included: hMPP7-F: 5’-CCTTCCTCTGGGATATGTTTGGT-3’ and hMPP7-R: 5’-AGCCTTCACATTGGGTTTTGA-3’. The primers for hGAPDH were designed as follows: forward sequence 5’-CCACCCATGGCAAATTCC-3’ and reverse sequence 5’-TGGGATTTCCATTGATGACAAG-3’. Target gene expression levels were standardized against hGAPDH reference values and calculated through the ΔΔCt analytical approach.

### Mice

All mouse experiments were performed in accordance with relevant guidelines and regulations of the Institutional Animal Care and Use Committee, and the study is reported in accordance with ARRIVE guidelines (https://arriveguidelines.org). MPP7 knockout (MPP7^−/−^) mice were purchased from Cyagen Biosciences and subsequently maintained under the same SPF vivarium conditions as WT-C57BL/6 controls at our institution. All HDM sensitization/challenge procedures, sample collection, and downstream assays were performed by the authors. Experimental protocols involving 6–8 week-old mice housed in specific pathogen-free environments received authorization. All mice (anesthetized by 3% isoflurane) were euthanized using CO_2_ overdose at the end of experiments. This study was reviewed and approved by the Animal Ethics Committee of Henan University of Chinese Medicine’s Zhengzhou People’s Hospital (Ethics No. 2025-KY-003801). All experiments were performed in accordance with relevant guidelines and regulations. The study was carried out in compliance with the ARRIVE guidelines (https://arriveguidelines.org).

### Allergen‑induced mouse asthma model

Female WT-C57BL/6 and MPP7^−/−^-C57BL/6 mice aged 6–8 weeks (20–25 g) were housed at 20–25℃ in a 12-h light/dark cycles condition. Mice were randomly divided into the WT PBS group, the WT HDM group, the MPP7^−/−^ PBS group, and the MPP7^−/−^ HDM group (six mice/group). Mice were exposed to house dust mite (HDM) extract (15 µg of protein in 10 µL PBS, Greer lads, Lenoir, NC, USA) intratracheally under isoflurane anesthesia on days 0 and 7. Mice in the control group received 10 µL sterile PBS intratracheally. Subsequent daily challenges with 20 µg HDM preparations occurred from days 14–18. Euthanasia performed 48 h post-final exposure enabled evaluation of airway inflammatory markers.

### Bronchoalveolar lavage fuid (BALF) analysis

The right lung was subjected to three sequential bronchoalveolar lavages using 0.5 mL of pre-warmed (37 ℃) PBS to improve improve procedural consistency and sample recovery. Collected BALF underwent centrifugation (500 × g, 10 min, 4 °C) for cellular separation. The resulting supernatants were aliquoted and cryopreserved at −80 °C until cytokine analysis.

### Quantifcation of HDM‑specifc IgE

Cardiac puncture-derived blood samples were permitted to coagulate for 30 min at ambient temperature prior to serum isolation through centrifugation (1000 × g, 10 min, 4 °C). Serum HDM-specific IgE quantification employed an adapted sandwich ELISA protocol (BioLegend, Cat:432404). Briefly, Nunc MaxiSorp plates (BioLegend) were incubated overnight at 4 °C with anti-mouse IgE coating antibodies. Following blocking procedures, serum specimens were applied for 2-hour incubation. Subsequent steps involved 1-hour exposure to biotin-conjugated HDM antigen (CITEQ) followed by HRP-streptavidin conjugation. TMB substrate facilitated 20-minute chromogenic development, with reaction termination achieved using 2 M sulfuric acid. Optical density measurements at 450 nm were recorded via Synergy H1 microplate reader (BioTek), with OD values directly correlating to HDM-specific IgE levels.

### ELISA

The levels of IL-4, IL-5, IL-6, TNF-α, and IL-13 were measured using sandwich ELISA kits obtained from BioLegend and Invitrogen, following the protocols provided by the manufacturers.

### Histopathological examination of the lungs

For histopathological analysis, left lung tissues were fixed in 4% phosphate-buffered formaldehyde for 24 h or longer before dehydration and paraffin embedding. Tissue sections (4 μm thickness) underwent haematoxylin & eosin (H&E) staining for inflammation assessment and periodic acid-Schiff (PAS) staining for mucus visualization, with observations conducted through a Nikon 80i microscope. A semi-quantitative scoring system was applied to inflammatory alterations: 0 indicated no infiltration; 1 represented minor cellular accumulation; 2 corresponded to a single-cell-layer inflammatory ring; 3 denoted two-to-four-cell-layer thickness; 4 indicated extensive infiltration exceeding four-cell layers.

### Immunofluorescence

For immunofluorescence staining, 6-µm cryosections were permeabilized in prechilled absolute methanol (− 20 °C) for 5 min and rinsed with Tris-buffered saline (TBS) containing 0.1% Tween-20. Sections were incubated overnight at 4 °C with the following primary antibodies: goat anti-CD11c (Servicebio, China, GB11059, 1:100), goat anti-CD117 (Bioss, China, bs-20717R, 1:100), goat anti-ECP (Proteintech, China, 55338-1-AP, 1:100), and rabbit anti-MPP7 (Proteintech, China, 12983-1-AP, 1:100). After washing in TBS containing 0.1% Tween-20, sections were incubated for 1 h at room temperature with donkey anti-goat Texas Red (Abcam, UK, ab7123, 1:1000) and donkey anti-rabbit cy3 (Abcam, UK, ab6939, 1:1000). Nuclei were counterstained with DAPI (Molecular Probes). Fluorescence images were acquired using a Leica laser-scanning confocal microscope. Images were collected at 16-bit depth using an HC PL FLUOTAR 20× objective at 160 nm/pixel. All channels were imaged with a 17 ms exposure time, 30% illumination intensity, and no binning. Z-stacks were acquired with 1-µm steps over a total depth of 20 μm.

### Generation and culture of BMDCs

Following deep sedation using 5% isoflurane and sterilization with 75% ethanol, mice were humanely sacrificed. Bone marrow from femora and tibiae was carefully harvested, after which muscle and connective tissues were removed through thorough cleaning. The bones were subsequently rinsed with chilled RPMI-1640 medium employing 25-gauge needles. Following erythrocyte removal using RBC lysis buffer (BioLegend), isolated marrow cells were resuspended at 1 × 10^6^ cells/mL in supplemented RPMI-1640 containing 10% fetal bovine serum, penicillin (100 U/mL), and streptomycin (100 µg/mL). Cell suspensions were cultured under standard conditions (37 °C, 5% CO₂) with differentiation factors including 20 ng/mL GM-CSF and 10 ng/mL IL-4 (both from R&D Systems). Partial medium replacement occurred on day 3, involving removal of non-adherent populations and 75% of supernatant, followed by addition of fresh cytokine-enriched medium. Subsequent medium renewal (50% volume) was performed on day 5. After seven days of culture, non-adherent immature BMDCs were collected, while parallel cultures underwent final maturation through 24-hour LPS exposure (100 ng/mL) with concurrent half-medium replacement.

### Isolation of lung DCs

Lung tissues were dissected, cut into small pieces (1 mm³), and subjected to enzymatic digestion in RPMI 1640-based buffer containing 1 mg/mL collagenase IV and 200 U/mL DNase I. The mixture was maintained at 37 °C with continuous agitation (250 rpm) for 45 min. Following digestion, cellular suspensions were filtered through a sterile 70 μm mesh. Erythrocytes were eliminated via hypotonic lysis, and lung-derived cells were pelleted via centrifugation. CD11c-expressing dendritic cells were purified through positive immunomagnetic sorting with UltraPure MicroBeads (Miltenyi Biotec) using LS column technology per manufacturer protocols, routinely achieving over 95% CD11c-positive populations. Cellular characterization was performed using flow cytometry.

### Flow cytometric analysis

Single-cell suspensions were generated following established protocols and diluted to achieve a density of 1 × 10^7^ cells/mL. Aliquots of 100 µL cell suspension were distributed into flow cytometry tubes, with prior administration of Fc-receptor blocking reagent (anti-mouse CD16/CD32, BD Biosciences) to reduce nonspecific interactions. Cellular samples underwent staining with fluorescence-labeled monoclonal antibodies targeting specific surface markers: CD11c-BUV395 (HL3), MHC II-FITC (2G9), CD45-BV510 (30-F11), CD11b-PerCP-Cy5.5 (M1/70), CD103-PE (2E7), CD86-BV421 (GL-1), and CD40-APC (3/23), along with appropriate isotype-matched controls (BD Biosciences) as specified by the supplier. Following a 30-minute dark incubation period at 4 °C, cells underwent single-wash centrifugation with FACS buffer before prompt analysis. Flow cytometric measurements were conducted using an LSRFortessa X-20 system (BD Biosciences), with subsequent data processing executed through FlowJo analytical software (v10.8).

Cells were gated by FSC/SSC, followed by singlet selection (FSC-A vs. FSC-W) and subsequent identification of CD45^+^ leukocyte. Lung DCs were identified as CD11c^+^MHC II^+^ cells, and subsets were defined by CD11b and CD103 expression (Fig. S2).

For BMDCs analyses, events were gated by FSC/SSC, followed by singlet selection (FSC-A vs. FSC-H) and live-cell gating prior to quantification of CD86 and CD40 expression (Fig. S3).

### Quantitative reverse transcription polymerase chain reaction (RT‑qPCR)

Total RNA extraction was conducted from either whole-lung tissue samples or fluorescence-activated cell-sorted pulmonary dendritic cells with TRIzol reagent (Invitrogen), followed by reverse transcription employing the RevertAid First-Strand cDNA Synthesis Kit (Thermo Fisher Scientific). Gene amplification was carried out through quantitative PCR with SYBR Green Master Mix (TOYOBO) on Roche’s LightCycler 96 system. The primer sequences employed in the study included: mMPP7-F: 5’-AGCTGAGGGAAGTGAATGGAAGT-3’, mMPP7-R: 5’-TGCCTC TTTACACGGGATTGC-3’; mIL-6-F: 5’-GGCGGATCG GATGTTGTGATGT-3’, mIL-6-R: 5’-GGACCCCAGACAATCGGTTG − 3’; mTNF-α-F: 5’-ACCCTCACACT CACAAACCACGT-3’, mTNF-α-R: 5’-ATAG CAAATCGGCTGACGGT-3’; mIL-12-F: 5’-CACACTGGACCAAAGGGACTGT − 3’, mIL-12-R: 5’-TGATGAAG AAGCTGGTGCTG-3’; mGAPDH-F: 5’-TGCCCAGAACATCATCCCT-3’, mGAPDH-R: 5’-GGTCCTCAGTGTAGCCCAAG-3’. Gene expression levels were standardized against mGAPDH as an internal control and analyzed through the ΔΔCt calculation approach.

### Western blotting

BMDCs were stimulated with LPS (100 ng/mL) for varying durations (0–120 min). After treatment, cells were collected and lysed using chilled RIPA buffer containing both protease and phosphatase inhibitors. Protein samples underwent electrophoresis on 12% SDS-PAGE gels followed by transfer to PVDF membranes (Millipore). Immunoblotting was performed using primary antibodies from Cell Signaling Technology specific for phosphorylated and total forms of AKT, NF-κB p65, and PI3K, along with GAPDH, with overnight incubation at 4 °C. Chemiluminescent detection was carried out using enhanced substrate reagents, and imaging was conducted with the Amersham Imager 600 system (GE Healthcare).

### RNA extraction and RNA-sequencing (RNA-Seq) assay

RNA sequencing-based transcriptomic analysis was performed by BGI Genomics Co., Ltd. DCs underwent LPS stimulation (100 ng/mL) over a 24-hour period. Total RNA isolation from DCs subjected to varying treatments was carried out with TRIzol reagent (Invitrogen). DCs without treatment were designated as controls. DESeq2 (version 1.34.0) was employed to detect differentially expressed genes (DEGs) distinguishing the experimental and control groups. Functional enrichment assessments of DEGs, including Gene Ontology (GO) and Kyoto Encyclopedia of Genes and Genomes (KEGG) pathway analyses, were conducted through the Biotools RNA-Seq platform (version 1.6.8).

### Statistical analysis

Group comparisons were preceded by tests of normality (Shapiro–Wilk) and variance homogeneity (Levene’s test) to ensure appropriate use of one-way ANOVA or non-parametric Kruskal–Wallis, with Fisher’s LSD or Games–Howell post hoc analysis (for ANOVA) and Dunnett’s multiple-comparison method (for non-parametric tests) applied as appropriate. For transcriptomic analysis, DESeq2 was used to identify differentially expressed genes, with significance defined at an adjusted P-value (false discovery rate) < 0.05, and downstream GO/KEGG enrichment analyses were performed on the genes meeting this FDR threshold.

##  Results

### Decreased MPP7 expression in the peripheral blood of asthmatic patients and the lungs of HDM induced asthma models

**Fig. 1:**
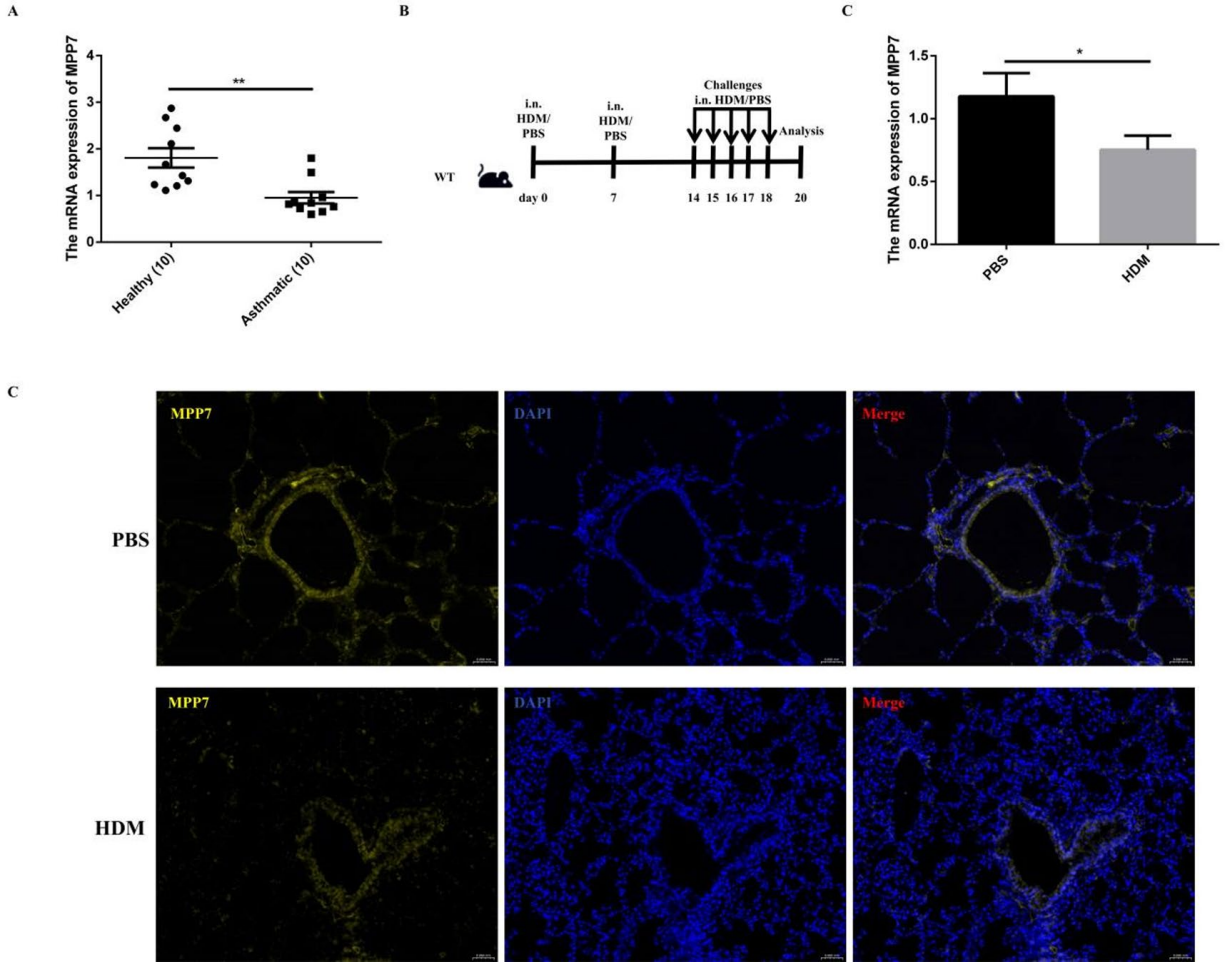
Asthma-associated reduction in MPP7 expression in human and murine subjects. A, qPCR analysis of MPP7 in peripheral blood from healthy (*n* = 10) and asthmatic (*n* = 10) individuals. B, HDM sensitization/challenge protocol for wild type mice. Analysis occurred 2 days postfnal challenge. C, qPCR analysis of MPP7 in lung tissues from control and HDM-exposed mice. D, Immunofluorescence of lung sections from control and HDM-exposed mice. *P < 0.05, **P < 0.01.

In this investigation, initial analyses focused on measuring MPP7 expression levels in peripheral blood of asthmatic individuals. Comparative data presented in Fig. 1A demonstrate substantial decreases in MPP7 concentrations among asthma patients compared to non-asthmatic subjects. Subsequent experimental approaches evaluated how HDM sensitization and exposure influenced pulmonary MPP7 expression patterns using a murine allergic asthma model (Fig. 1B). Figure 1C shows reduced MPP7 mRNA levels in whole-lung homogenates from HDM-exposed mice compared with PBS controls. Cell-type/spatial localization was assessed by immunofluorescence in lung sections (Fig. 1D), which demonstrates MPP7 signal within airway-associated structures and reduced staining intensity after HDM challenge. In addition, immunofluorescence of PBS-exposed mice lung tissue revealed MPP7 displayed high expression in DCs but showed negligible levels in mast cells and eosinophils, as shown in Figure S1. Together, these data indicate that reduced MPP7 expression is associated with HDM-driven allergic airway inflammation and may represent a potential biomarker candidate, pending validation in larger, clinically phenotyped cohorts and in other airway inflammatory conditions.

#### Augmented HDM‑induced allergic airway infammation in MPP7 defcient mice

**Fig. 2:**
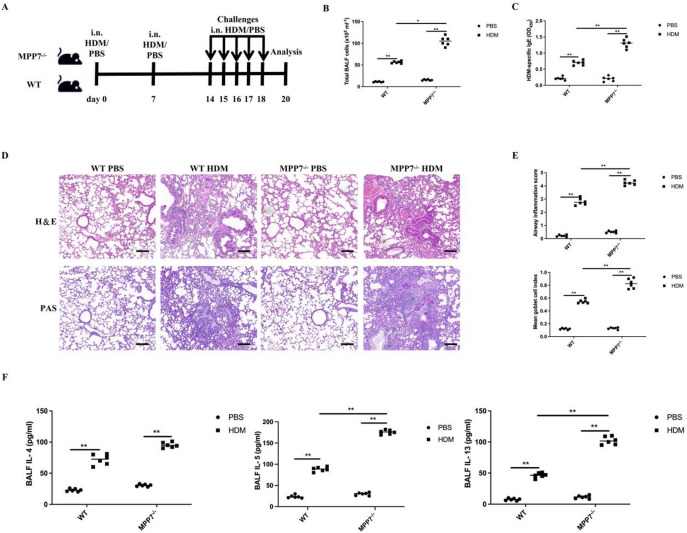
Enhanced allergic asthma in MPP7^−/−^ mice post HDM exposure. **A** HDM sensitization/challenge protocol for wild type and MPP7^−/−^ mice, with PBS controls. Analysis occurred 2 days postfnal challenge. **B** BALF cell counts (*n* = 6/group). **C** ELISA quantifcation of HDM-specifc IgE (*n* = 6/group). **D** Lung histology stained with H&E or PAS; scale bar = 200 μm. **E** Quantitative assessment of infammatory infltration and goblet cell hyperplasia (*n* = 6/group). **F** ELISA determination of IL-4, IL-5, and IL-13 in BALF (*n* = 6/group). Data are mean ± SEM. *P < 0.05, **P < 0.01.

To investigate the role of MPP7 in protecting against allergen-induced pulmonary pathology, we created MPP7-deficient (MPP7^−/−^) murine models. As shown in Fig. 2A, both MPP7^−/−^ and wild-type (WT) mice underwent intranasal HDM sensitization and subsequent repeated allergen exposures. Analysis of BALF demonstrated that HDM-treated MPP7^−/−^ and WT groups exhibited a notable increase in inflammatory cell counts compared to vehicle-treated counterparts (Fig. 2B). Notably, MPP7^−/−^ animals displayed significantly higher serum concentrations of HDM-specific IgE relative to HDM-exposed WT controls (Fig. 2C).

Histopathological examination of HDM-treated MPP7^−/−^ mice showed greater bronchial wall thickening, intensified peribronchial inflammatory cell accumulation, and heightened PAS-positive goblet cell proliferation compared to controls (Fig. 2D, E). Concurrently, elevated concentrations of Th2-associated cytokines (IL-4, IL-5, and IL-13) were detected in the BALF of HDM-challenged MPP7^−/−^ mice relative to WT counterparts (Fig. 2F). These findings collectively indicate enhanced allergic airway responses in the absence of MPP7.

The absence of MPP7 was shown to intensify Th2-mediated immunological reactions and elevate mucus secretion in response to HDM challenge. These results demonstrate MPP7’s essential role in suppressing allergen-driven inflammatory processes within pulmonary tissues.

#### MPP7 modulates the phenotype and function of lung DCs in asthmatic mice

**Fig. 3:**
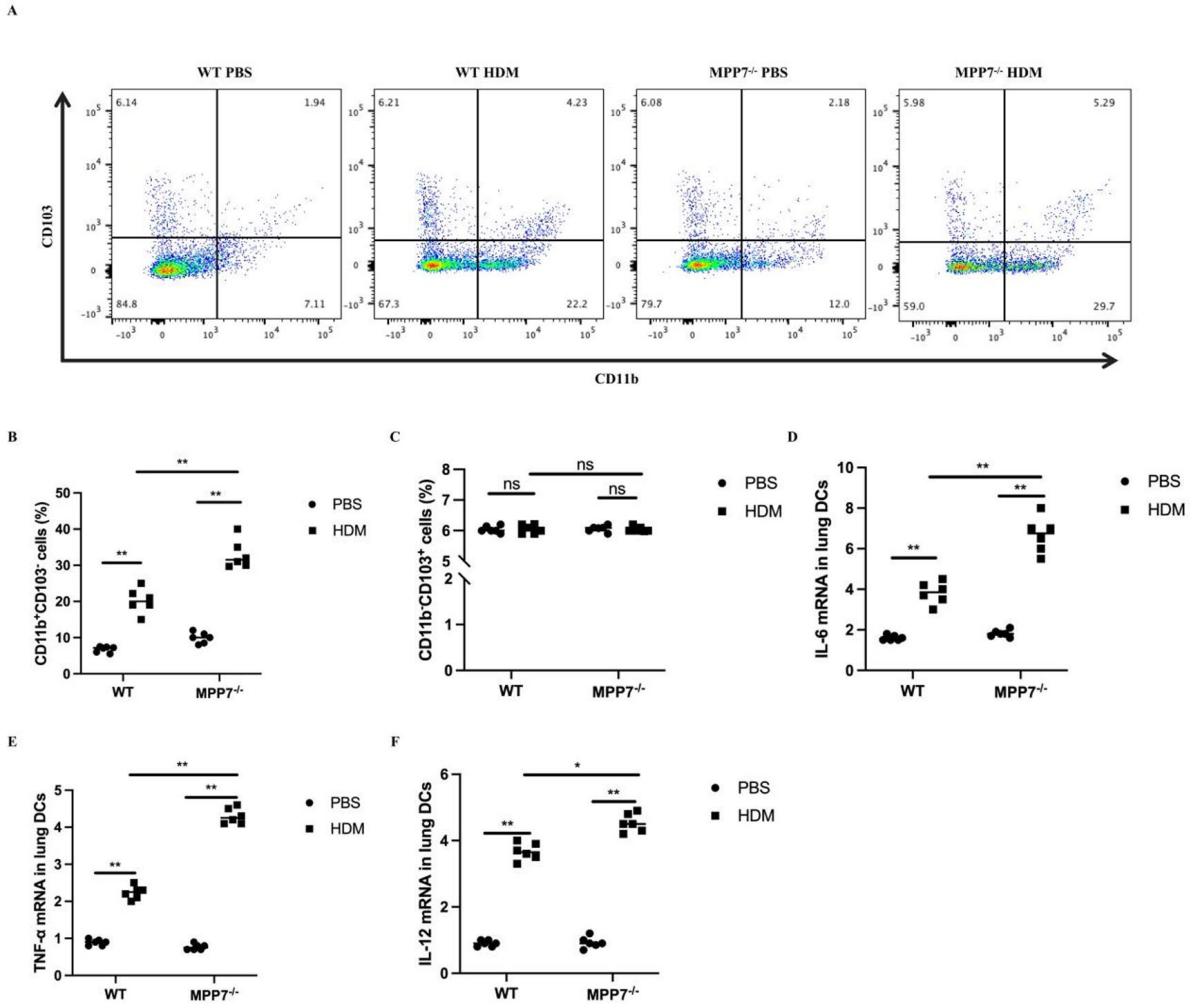
Augmented DCs activation and Th2 expansion in MPP7^−/−^ mice post-HDM challenge. **A** Flow cytometry of lung CD11b^+^CD103^−^ and CD11b^−^CD103^+^ DCs. **B** and **C** Quantifcation of DC subsets (*n* = 6/group). **D**–**F** qPCR analysis of IL-6, TNF-α, IL-12 mRNA levels in lung DCs post-HDM exposure (*n* = 3/group). *P < 0.05, **P < 0.01, ns not signifcant.

Due to the high expression of MPP7 in DCs, subsequent analysis focused on comparing pulmonary DC subpopulations and activation states between WT and MPP7-deficient mice during HDM-triggered allergic airway inflammation. Analyses using flow cytometry demonstrated a substantial elevation of CD11b^+^CD103^−^ conventional type 2 DCs (cDC2s) in HDM-exposed MPP7^−/−^ lungs, while CD11b^−^CD103^+^ conventional type 1 DCs (cDC1s) showed no significant differences compared to WT counterparts (Fig. 3A-C). Quantitative PCR analysis revealed that allergen exposure induced upregulated expression of IL-6, TNF-α, and IL-12 mRNA in pulmonary DCs from both genotypes, though these increases were more pronounced in MPP7-deficient mice relative to WT controls (Fig. 3D-F).

#### MPP7 suppresses immune activation in BMDCs

**Fig. 4:**
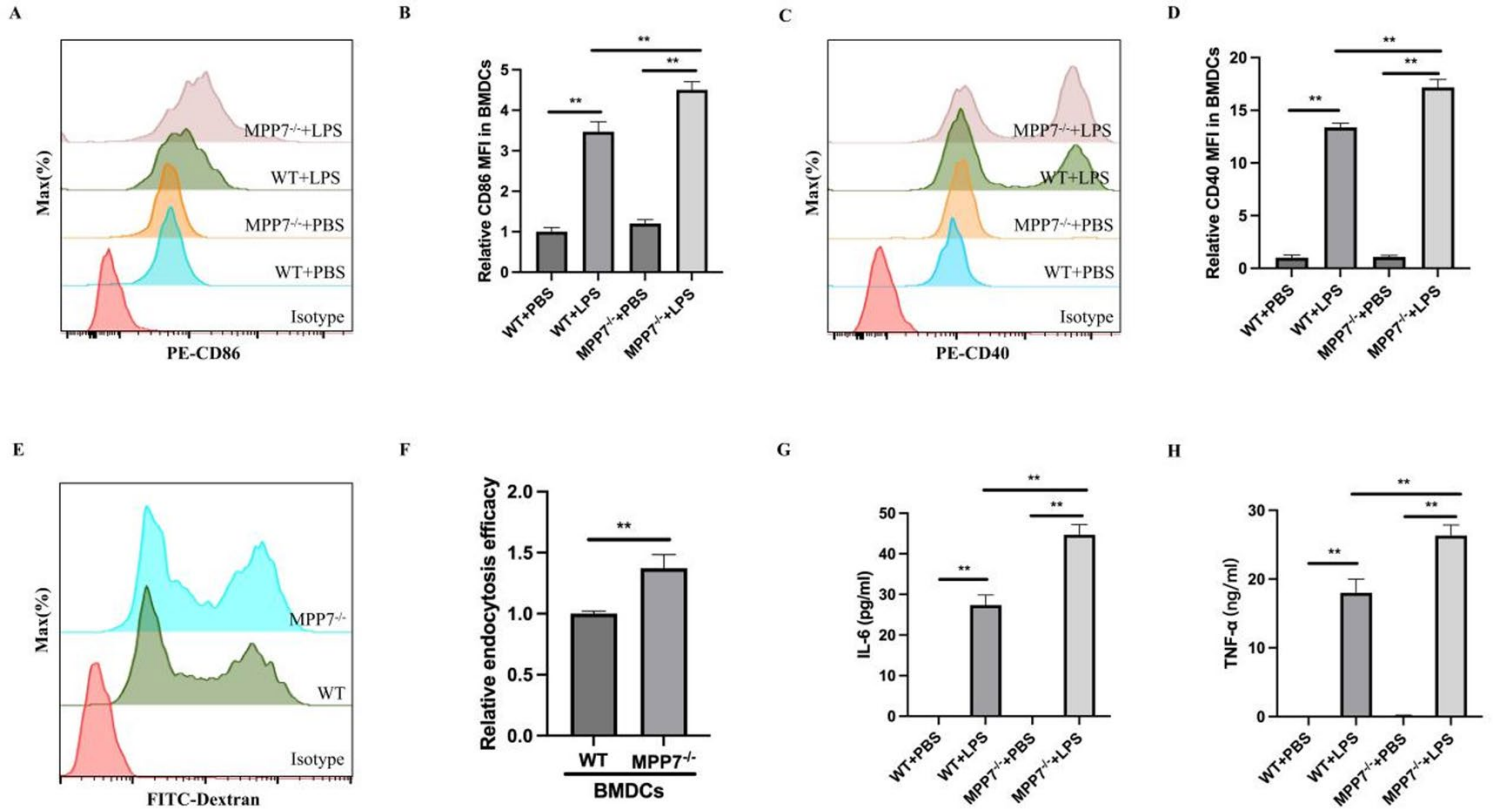
MPP7 modulates immunological activity of BMDCs In Vitro. A-D, Flow cytometric analysis of CD86 and CD40 on MPP7^−/−^ BMDCs post LPS (100 ng/mL) treatment. E and F, FITC-Dextran endocytosis by immature BMDCs. G and H, ELISA for cytokines in supernatants of LPS-stimulated MPP7^−/−^ BMDCs (*n* = 3/group). All data are shown as mean ± SEM. *P < 0.05, **P < 0.01.

To investigate MPP7’s direct regulatory effects on DCs functionality, we utilized an in vitro BMDCs culture model. LPS stimulation was used as a standardized in vitro dendritic-cell maturation stimulus to test intrinsic effects of MPP7 on DC activation and signaling. LPS treatment triggered substantial elevation in surface markers CD86 and CD40 – key mediators of T-cell activation (Fig. 4A-D). The treatment additionally provoked marked increases in pro-inflammatory cytokine production, particularly IL-6 and TNF-α (Fig. 4G, H). Notably, BMDCs lacking MPP7 showed amplified surface expression of these co-stimulatory molecules compared to wild-type counterparts under LPS stimulation (Fig. 4A-D). Unstimulated MPP7^−/−^ BMDCs displayed greater FITC-dextran internalization than WT cells, suggesting heightened endocytic activity (Fig. 4E, F). Moreover, MPP7-deficient cells produced elevated quantities of IL-6 and TNF-α post-LPS exposure relative to control groups (Fig. 4G, H).

#### Altered transcriptomic profile in MPP7^−/−^ LPS-treated BMDCs

**Fig. 5:**
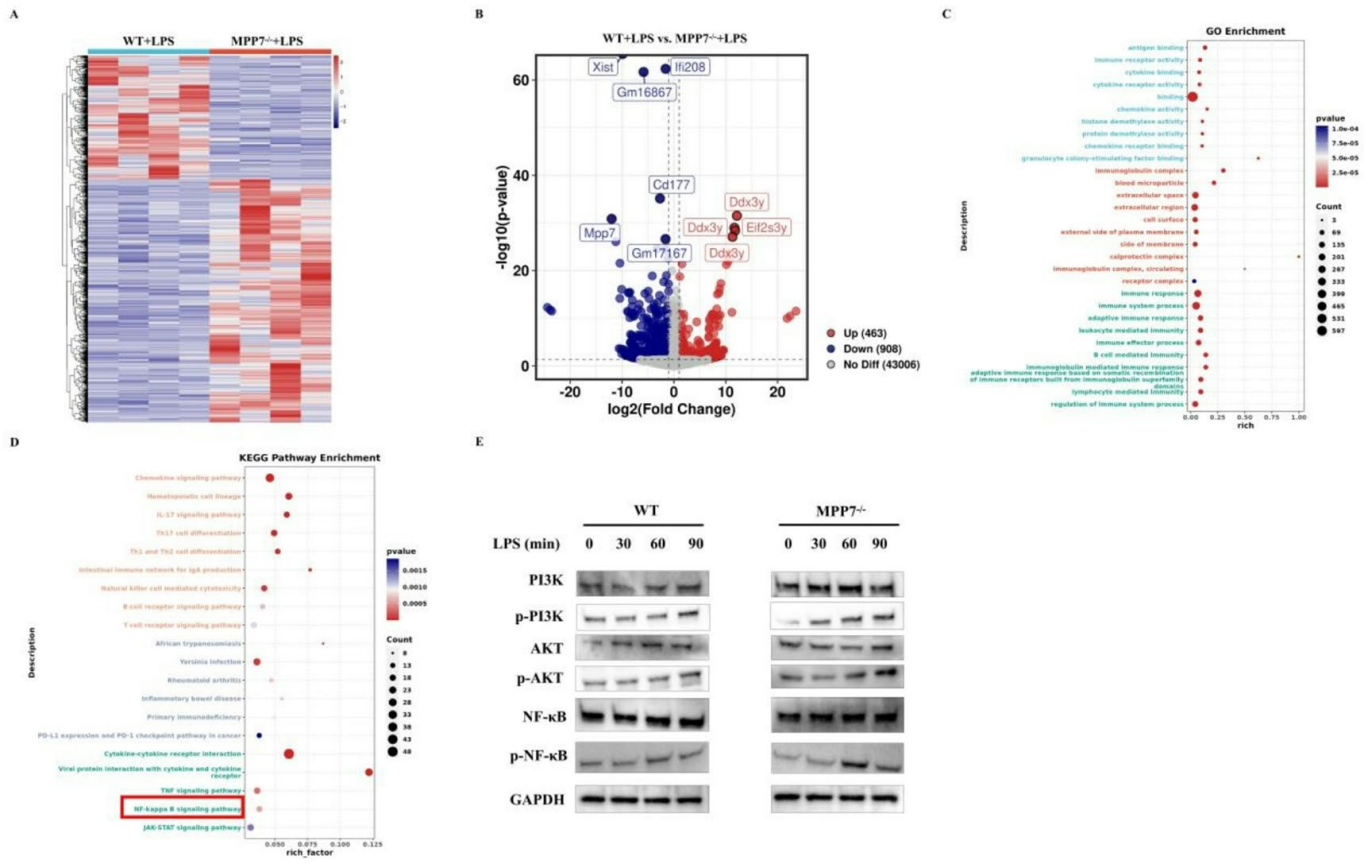
Comparative transcriptomic analysis between LPS-activated WT BMDCs and MPP7^−/−^ BMDCs. **A** A heatmap of DEGs among the WT + LPS group and MPP7^−/−^+LPS group. Red and blue respectively represent upregulated and downregulated genes. **B** Volcano plot of DEGs between the WT + LPS and MPP7^−/−^+LPS groups. **C** GO enrichment analysis of DEGs between the WT + LPS and MPP7^−/−^+LPS groups. **D** KEGG pathway enrichment of DEGs was identified in the MPP7^−/−^+LPS group compared to the WT + LPS group. KEGG pathway enrichment analysis was performed using the KEGG database (Kanehisa Laboratories; www.kegg.jp/kegg/kegg1.html). **E** Western blot analysis of PI3K, AKT, and NF-κB phosphorylation in WT + LPS and MPP7^−/−^+LPS groups. GAPDH used as loading control.

To investigate the molecular pathways through which MPP7 influences DCs biology, bulk RNA-seq was performed on four experimental conditions: WT + PBS, WT + LPS, MPP7^−/−^+PBS, and MPP7^−/−^+LPS. Figure 5A illustrates a heatmap visualizing DEGs between WT + LPS and MPP7^−/−^+LPS groups. Comparative analysis using volcano plots of WT + LPS versus MPP7^−/−^+LPS BMDCs revealed 463 upregulated and 908 downregulated transcripts (Fig. 5B). GO assessment of these DEGs indicated that MPP7 knockout primarily altered biological processes linked to immune receptor functions, cytokine receptor interactions, and cytokine-mediated activities (Fig. 5C). Subsequent KEGG pathway evaluation showed significantly enriched pathways were primarily involved in TNF signaling, IL-17 signaling, and NF-κB activation cascades (Fig. 5D). Building on existing research connecting the PI3K-AKT-NF-κB axis to asthma-related inflammation, subsequent investigations focused on phosphorylation patterns in these signaling networks. Within this signaling pathway component, biochemical assays demonstrated that loss of MPP7 function increased phosphorylation levels of PI3K, AKT, and NF-κB signaling molecules (Fig. 5E). Combined with transcriptional profiling evidence, these findings suggest MPP7 acts as a suppressor of DCs maturation and activation processes, playing an immunomodulatory role through these mechanisms.

##  Discussion

While previous studies have associated MPP7 with respiratory disease development, its molecular mechanisms remain partially characterized. Our investigation measured MPP7 concentrations in asthmatic patients’ peripheral blood while simultaneously evaluating pulmonary expression patterns in experimental murine models of HDM-induced allergic airway inflammation. Both clinical and preclinical analyses consistently showed marked reduction in MPP7 expression, suggesting its utility as a potential biomarker for tracking allergic inflammatory responses in respiratory tissues.

To investigate the specific role of MPP7 in allergic airway inflammation pathogenesis, HDM-sensitized MPP7^−/−^ mice were analyzed alongside their WT counterparts. Consistent with expectations, MPP7-deficient animals displayed marked Th2-biased immune reactions. These responses were manifested through heightened inflammatory cell numbers in BALF, along with elevated levels of IL-4, IL-5, and IL-13 in BALF. Additionally, serum concentrations of HDM-specific IgE were significantly increased. Histopathological analysis revealed substantial peribronchial inflammatory cell accumulation, goblet cell proliferation, and excessive mucus production. Taken together, these findings indicate that MPP7 plays a crucial protective function in mitigating allergen-induced airway inflammation and the corresponding activation of Th2 lymphocytes.

DCs serve as the primary antigen-presenting cells, playing a distinct role in activating naïve T lymphocytes and triggering the initial stages of adaptive immune responses. This functional capacity enables them to link innate defenses with adaptive mechanisms during pulmonary inflammatory processes^16,17^. In airway structures, DC populations residing adjacent to or embedded within epithelial layers crucially determine T-cell differentiation patterns via sustained crosstalk with respiratory epithelial cells^18^. Notably, MPP7 exhibits strategic positioning that facilitates direct engagement with DC populations, potentially regulating their immunological activity. To investigate MPP7’s regulatory effects on pulmonary DC functionality in asthmatic conditions, we examined lung DC subpopulations using the HDM-induced MPP7-deficient murine model. The murine pulmonary DC compartment consists of two principal subtypes: plasmacytoid DCs (pDCs) and conventional/myeloid DCs (cDCs). The latter subsets are subdivided into CD11b^+^CD103^−^ cDC2 and CD103^+^CD11b^−^ cDC1 populations. cDC2s demonstrate enhanced capacity for releasing pro-inflammatory chemokines, driving Th2 cell differentiation, and intensifying downstream Th2-mediated immune reactions^19–21^. Following allergen exposure, MPP7-deficient mice showed a marked increase in cDC2 population size relative to WT counterparts. This collective evidence indicates MPP7 suppresses Th2-biased inflammatory responses during allergic reactions by limiting cDC2 expansion, thus revealing a critical regulatory pathway through which MPP7 influences asthma development mechanisms.

After capturing airborne antigens, DCs in the lungs travel to draining lymph nodes and undergo maturation into an activated state. This developmental shift is marked by elevated MHC-II levels on their surface, improving their capacity to present antigens to T cells, along with heightened expression of co-stimulatory molecules required for full T lymphocyte activation. CD86 plays a dominant role among these molecules, binding to CD28 or CTLA-4 receptors on T lymphocytes and serving as a key driver of Th2 cell differentiation following allergen exposure^22^. Our investigation revealed that the absence of MPP7 substantially enhanced both CD86 and CD40 expression in LPS-activated bone marrow-derived dendritic cells. These data collectively indicate that MPP7 suppresses dendritic cell maturation through coordinated downregulation of CD86 and CD40, thereby revealing an additional pathway through which MPP7 restrains Th2-biased inflammatory responses in experimental asthma models.

To investigate the mechanisms underlying MPP7-mediated suppression of DCs maturation, RNA sequencing was conducted on bone marrow-derived DCs. GO analysis highlighted immune regulatory processes as the most prominently enriched terms. Subsequent KEGG pathway examination demonstrated significant enrichment in TNF, IL-17, and NF-κB signaling pathways. Experimental validation showed MPP7 effectively reduced phosphorylation levels of PI3K, AKT, and NF-κB components. These findings align with prior research indicating that pharmacological blockade of this signaling cascade alleviates asthma symptoms in experimental models, as NF-κB activation regulates multiple inflammatory mediators in pulmonary tissues affected by asthma^23^. Supporting earlier observations that MPP7 inhibits NF-κB to reduce particulate matter-induced damage, our results suggest that MPP7 alleviates allergen-induced airway inflammation through suppression of the PI3K/AKT/NF-κB signaling axis.

Notwithstanding these advances, our study harbors limitations inherent to designs. Our human sample size was small (*n* = 10 asthmatics), inherently limiting statistical power and biomarker conclusions. Larger, well-phenotyped cohorts will be essential to determine whether MPP7 has meaningful diagnostic or prognostic value in asthma. MPP7 was deleted in all cell types in our knockout model, the exacerbated allergic phenotype observed cannot be attributed solely to DC dysfunction. Future studies employing DC–specific MPP7 conditional knockout mice will be crucial to definitively establish the DC-intrinsic role of MPP7 in allergic airway inflammation. Notably, all murine experiments in this study were conducted using female mice only. This sex-specific study design is a limitation, as male mice may exhibit different allergic airway responses; future investigations should include both sexes to assess any potential sex-dependent effects of MPP7 in asthma models. Finally, BALF cellularity was quantified as total inflammatory cell counts without differential enumeration of eosinophils and neutrophils. Future studies will include cytospin-based differential counts and/or flow-cytometric profiling of BALF granulocyte subsets to refine inflammatory endotyping.

## Conclusion

**Fig. 6:**
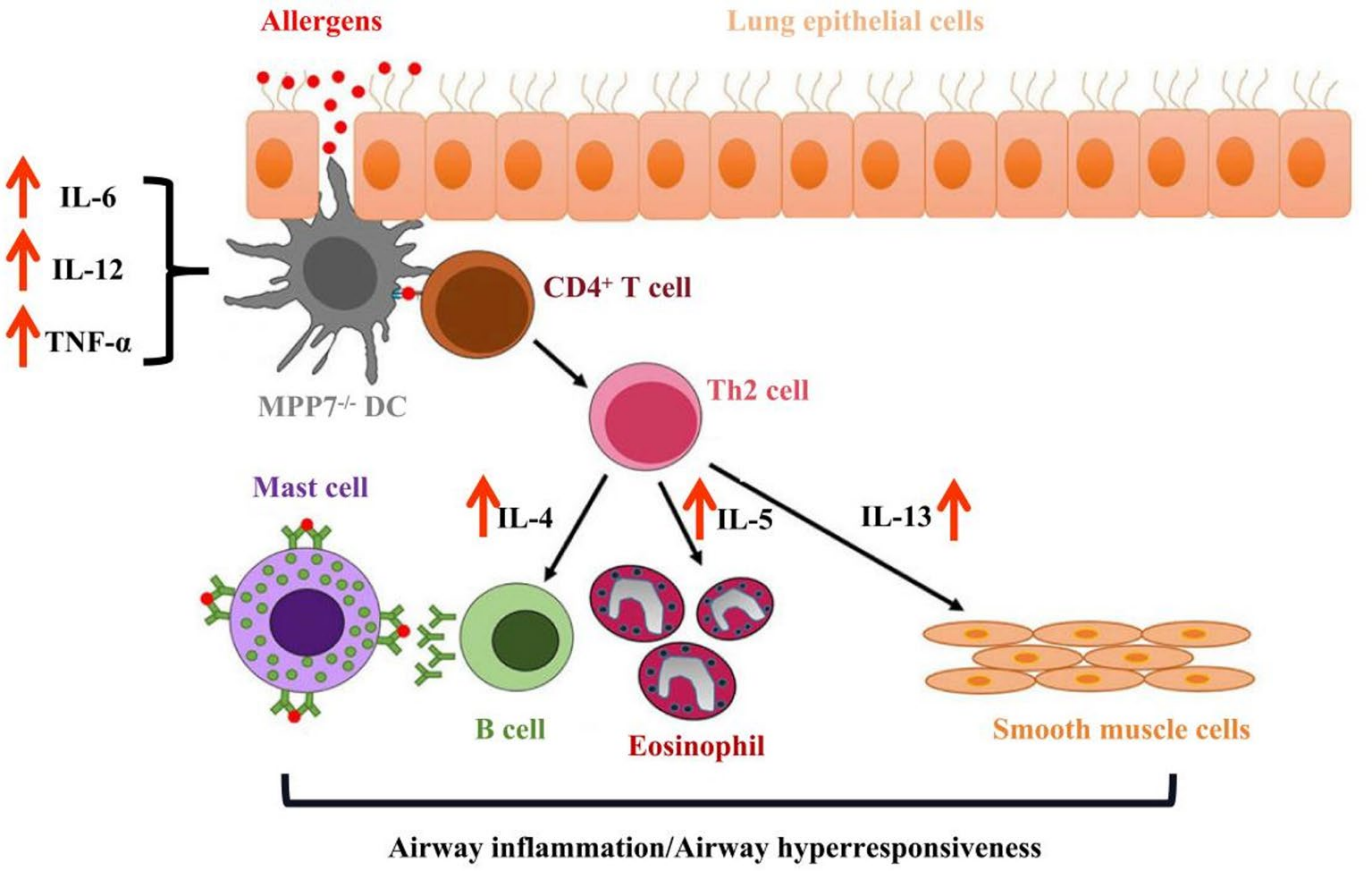
MPP7^−/−^ DCs aggravate Th2-mediated allergic airway inflammation. MPP7 indirectly attenuates Th2-polarised airway inflammation by reprogramming lung DC phenotype and function.

In summary, this study reveals markedly decreased MPP7 expression in asthmatic patients and experimental models. Targeted deletion of MPP7 in murine systems worsens HDM-triggered allergic airway pathology through amplified Th2 cytokine production, enhanced cellular infiltration, and elevated allergen-specific IgE levels. Mechanistically, MPP7 modifies the composition of pulmonary CD11b^+^CD103^−^ DCs subsets while limiting their ability to activate immune responses. These findings establish that MPP7 indirectly reduces type 2-skewed airway inflammation through functional and phenotypic modulation of lung dendritic cells (Fig. 6), suggesting its therapeutic promise for managing asthma and other respiratory inflammatory conditions.

## Supplementary Information

Below is the link to the electronic supplementary material.

Supplementary Material 1

Supplementary Material 2

Supplementary Material 3

Supplementary Material 4

Supplementary Material 5

Supplementary Material 6

Supplementary Material 7

Supplementary Material 8

Supplementary Material 9

Supplementary Material 10

Supplementary Material 11

Supplementary Material 12

Supplementary Material 13

Supplementary Material 14

Supplementary Material 15

Supplementary Material 16

Supplementary Material 17

Supplementary Material 18

Supplementary Material 19

Supplementary Material 20

Supplementary Material 21

Supplementary Material 22

## Data Availability

The RNA-seq datasets are available upon public access from the National Center for Biotechnology Information (NCBI, https://www.ncbi.nlm.nih.gov/), under accession numbers PRJNA1347929.

## Abbreviations

AMOT: Angiomotin
BMDCs: Bone-marrow-derived dendritic cells
BALF: Bronchoalveolar lavage fuid
CNTN1: Contactin-1
CREB: cAMP response element binding protein
cDC: Conventional/myeloid DCs
cDC1s: Conventional type 1 DCs
cDC2s: Conventional type 2 DCs
DCs: Dendritic cells
Dlg1: Discs large homolog 1
EGFR: Epidermal growth factor receptor
FITC: Fluoresceinisothiocyanate
FACS: Fluorescence-activated cell sorting
GINA: The Global Initiative for Asthma
GO: Gene Ontology
HDM: House-dust-mite
H&E: Haematoxylin & eosin
KEGG: Kyoto Encyclopedia of Genes and Genomes
MPP7: The MAGUK p55 subfamily member 7
MPP: The membrane palmitoylated protein
METRN-β: Meteorin-β
MARCH1: Membrane-associated RING-CH 1
pDCs: Plasmacytoid DCs
PAS: Periodic acid-Schiff
qPCR: Quantitative PCR
RNA-seq: RNA sequencing
RT‑qPCR: Quantitative reverse transcription polymerase chain reaction
TGF-β: Transforming growth factor β
Th2: T-helper-2
WHO: The World Health Organization
YAP1: Yes-associated protein
