# Supplementary material for: MAGUK p55 subfamily member 7 attenuates allergic airway inflammation by modulating lung dendritic cells functions
Source: Sci Rep. 2026 Feb 28;16:11473. doi: 10.1038/s41598-026-40491-w (PMC13057229; doi:10.1038/s41598-026-40491-w)
Supplement: Supplementary file 1 — Supplementary Material 1 [file 41598_2026_40491_MOESM1_ESM.docx]

**
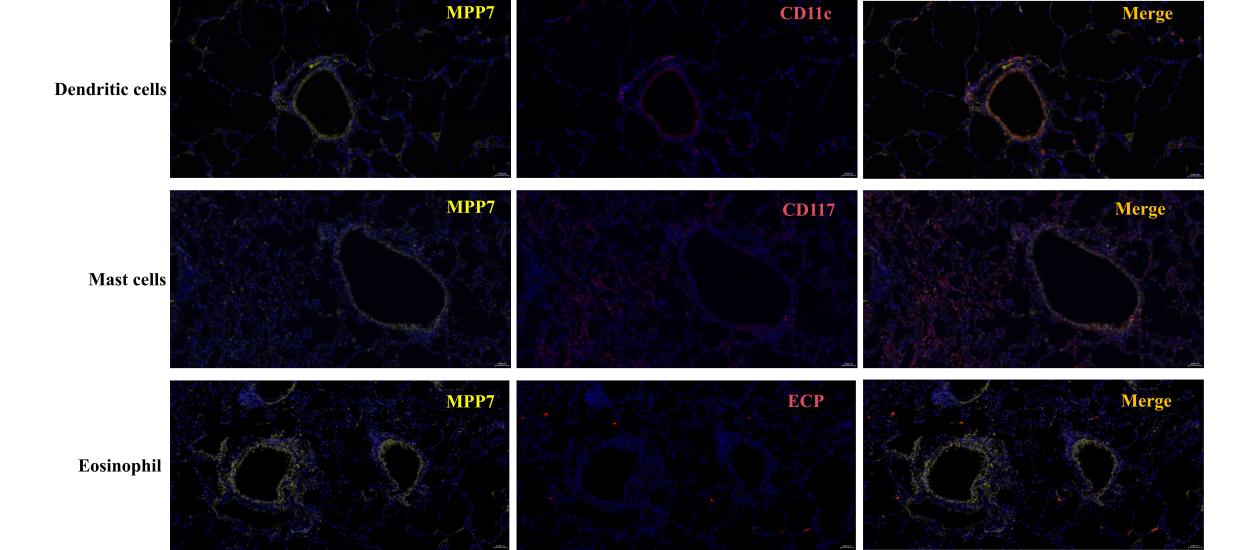
**

**Fig. S1 MPP7 expression in DCs, mast cells, and eosinophil of mice.** Immunofluorescence of lung sections from mice.


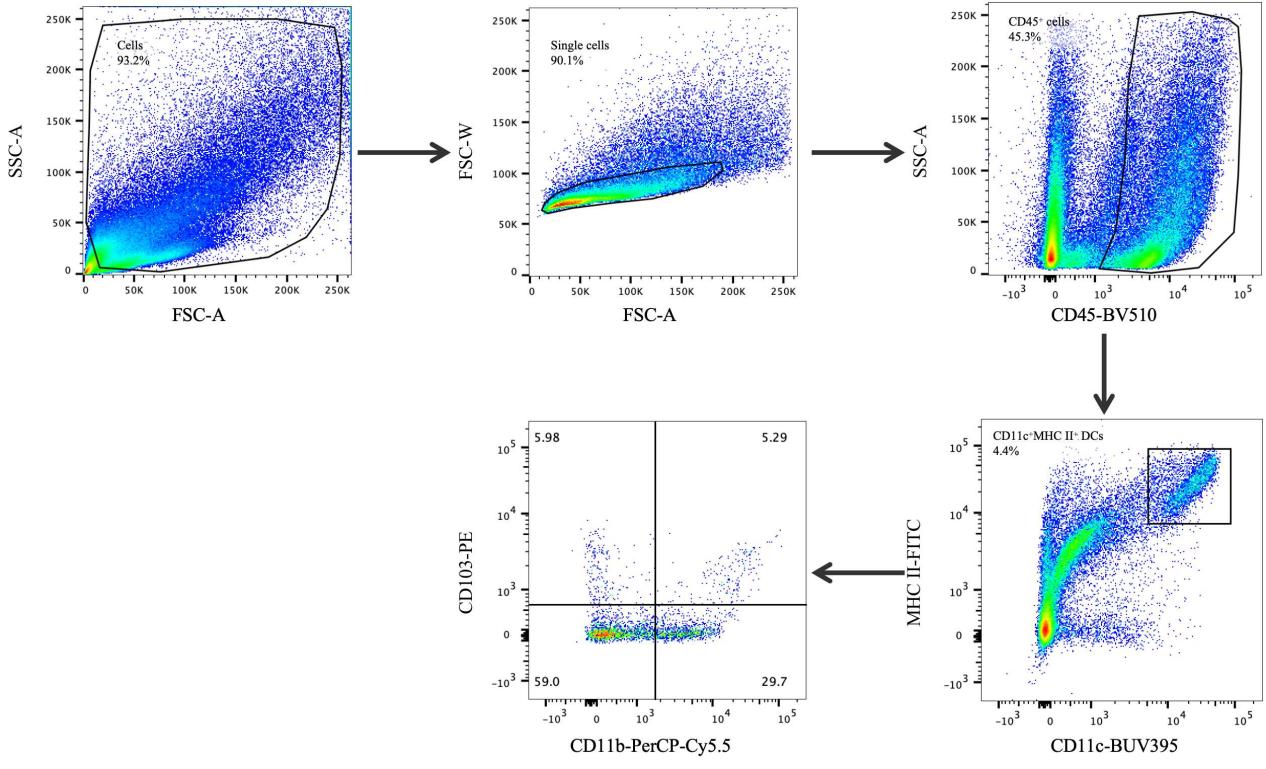


**Fig. S2** **Gating strategies for flow cytometric analyses.** Flow cytometry gating scheme for lung DCs subsets. DCs were identified as CD11c^+^MHC II^+^ cells and were gated out of singlet CD45^+^ cells. Gated DCs then analyzed by their expression of CD11b and CD103.


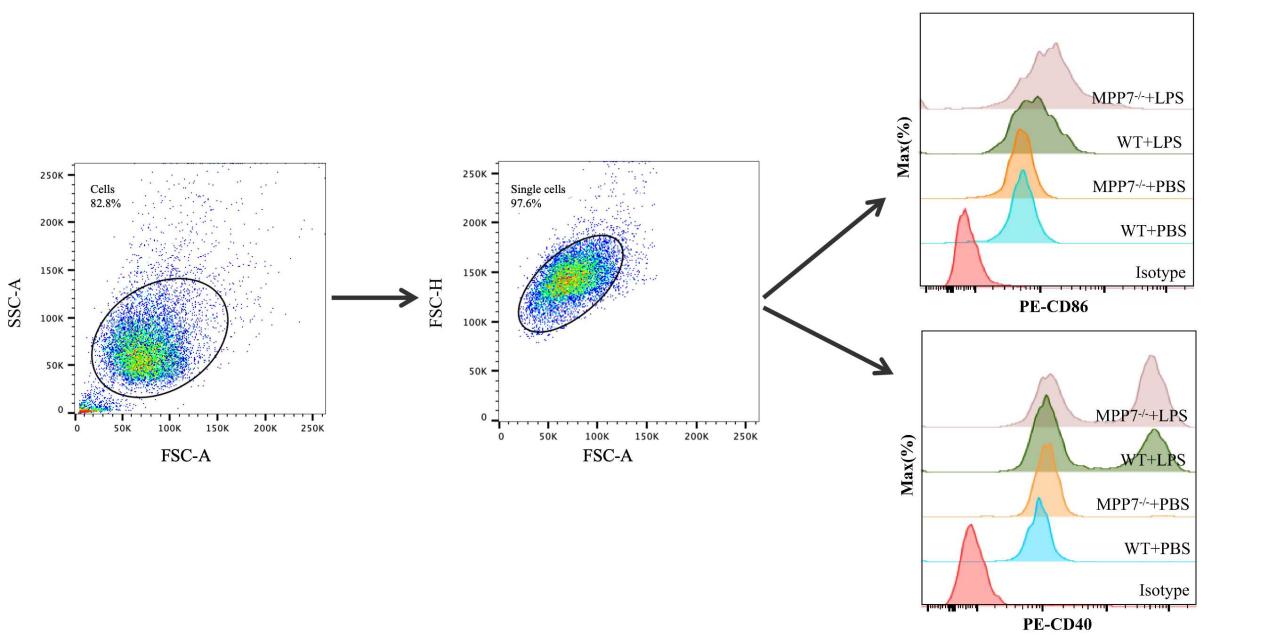
**Fig. S3 Gating strategies for flow cytometric analyses.** Flow cytometry gating scheme for BMDCs. BMDCs were gated by FSC/SSC, followed by singlet selection (FSC-A vs FSC-H) and live-cell gating prior to quantification of CD86 and CD40 expression.

**Table S1 The clinical characterization of asthmatic patients.**

| **Number** | **Sex** | **Age** | **IgE** | **Asthma severity** | **Medication use** |
| --- | --- | --- | --- | --- | --- |
| 92304001 | Male | 7 | 869.00 | Severe | Methylprednisolone sodium succinate, nebulized salbutamol, budesonide |
| 92350701 | Male | 10 | 203.00 | Severe | Methylprednisolone sodium succinate, nebulized salbutamol, budesonide, cefotaxime |
| 92338401 | Male | 9 | 453.00 | Severe | Methylprednisolone sodium succinate, nebulized salbutamol, budesonide |
| 92345301 | Male | 9 | 150.00 | Severe | Methylprednisolone sodium succinate, nebulized salbutamol, budesonide |
| 61581102 | Female | 7 | 329.00 | Severe | Methylprednisolone sodium succinate, nebulized salbutamol, budesonide, ceftriaxone |
| 91209604 | Female | 6 | 169.00 | Moderate | Methylprednisolone sodium succinate, nebulized salbutamol, budesonide, ceftriaxone |
| 64828401 | Female | 8 | 414.00 | Mild | Methylprednisolone sodium succinate, nebulized salbutamol, budesonide |
| 65435203 | Female | 5 | 982.00 | Moderate | Methylprednisolone sodium succinate, nebulized salbutamol, budesonide, ceftriaxone |
| 91907001 | Male | 10 | 2500.00 | Mild | Nebulized salbutamol, budesonide, ceftriaxone |
| 92065401 | Male | 6 | 869.00 | Mild | Methylprednisolone sodium succinate, nebulized salbutamol, budesonide |
