# Supplementary figures and images for: MAGUK p55 subfamily member 7 attenuates allergic airway inflammation by modulating lung dendritic cells functions
Source: Sci Rep. 2026 Feb 28;16:11473. doi: 10.1038/s41598-026-40491-w (PMC13057229; doi:10.1038/s41598-026-40491-w)

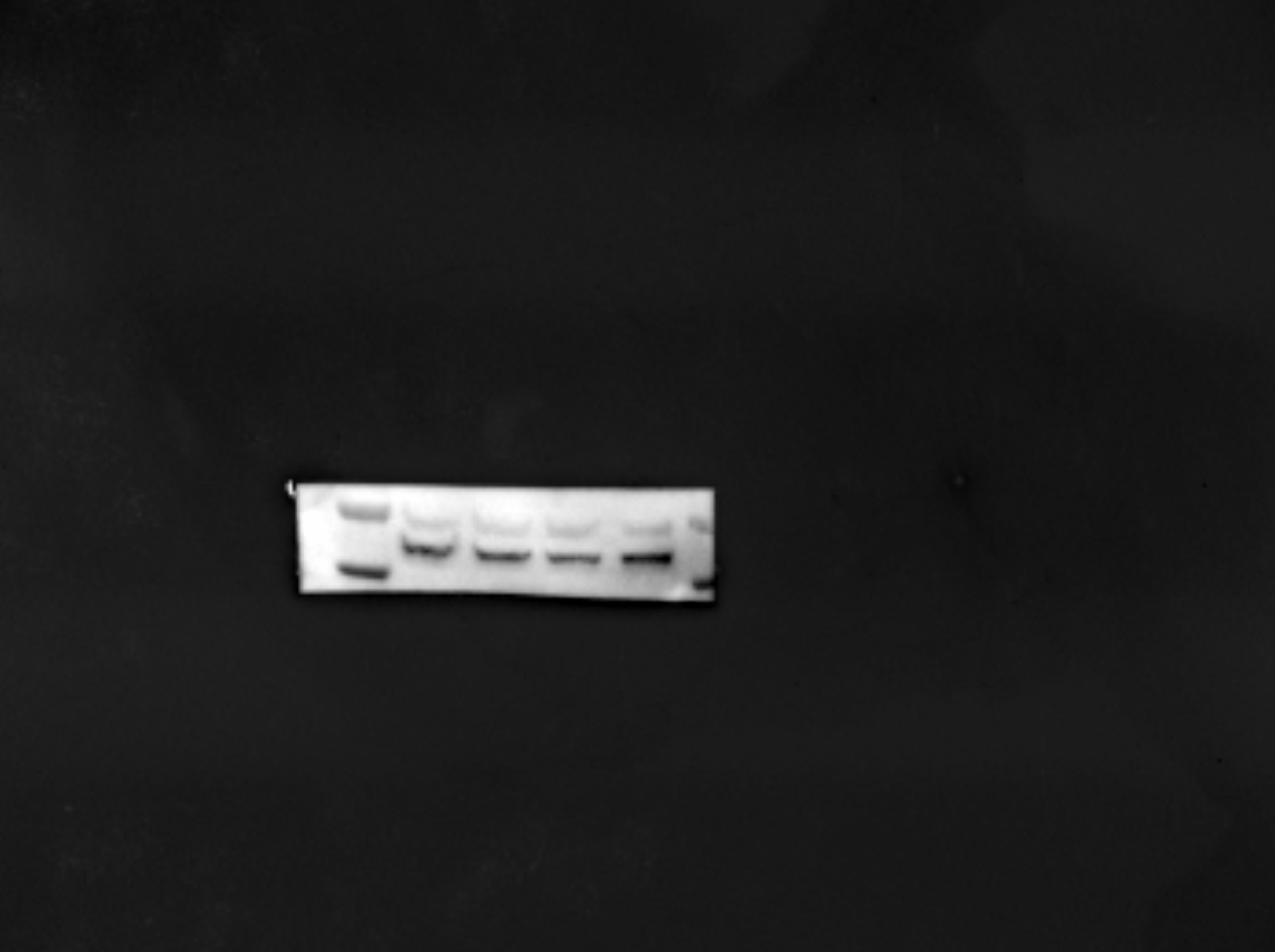

Supplement: Supplementary file 2 — Supplementary Material 2 [file 41598_2026_40491_MOESM2_ESM.tif]

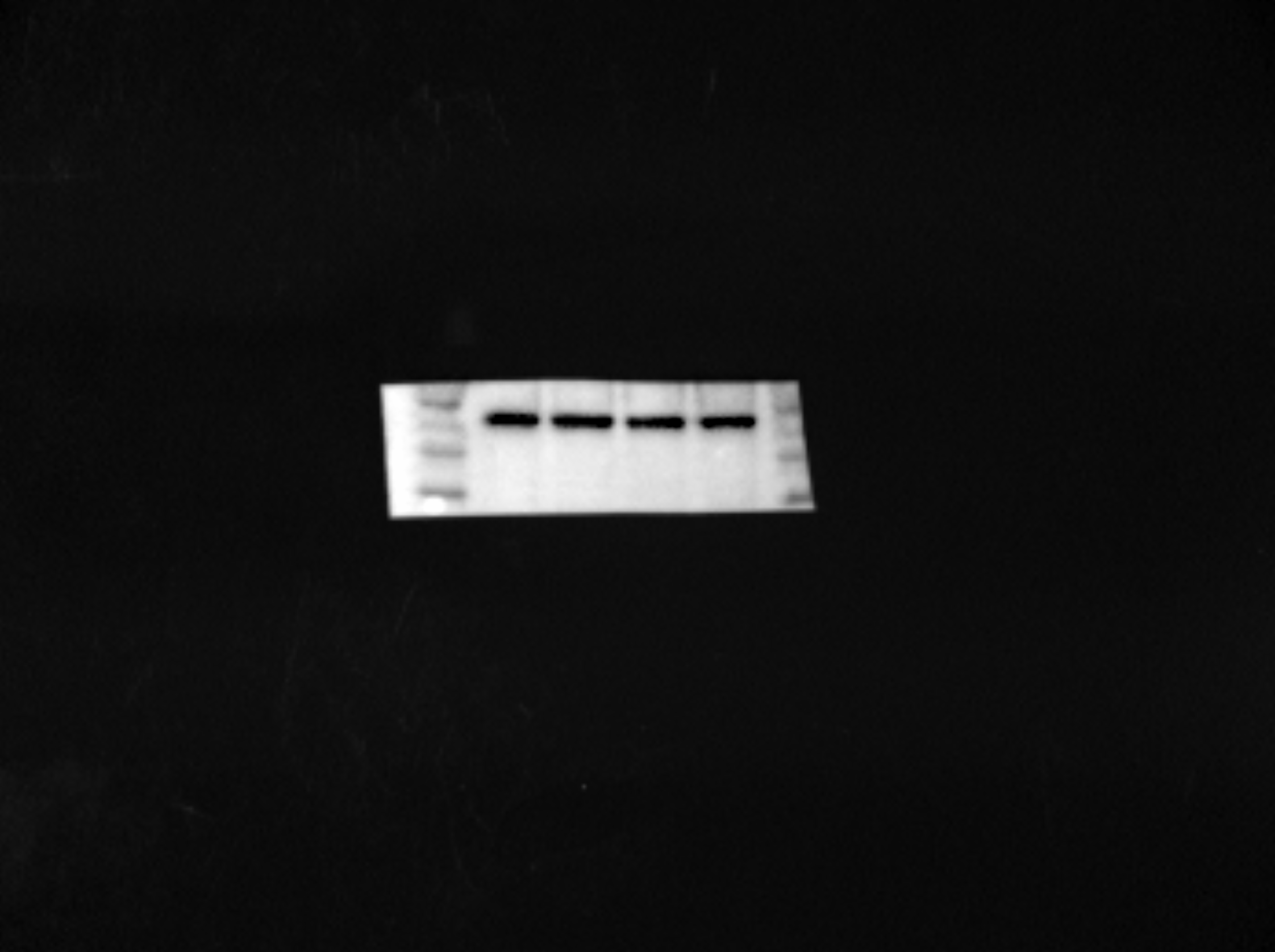

Supplement: Supplementary file 3 — Supplementary Material 3 [file 41598_2026_40491_MOESM3_ESM.tif]

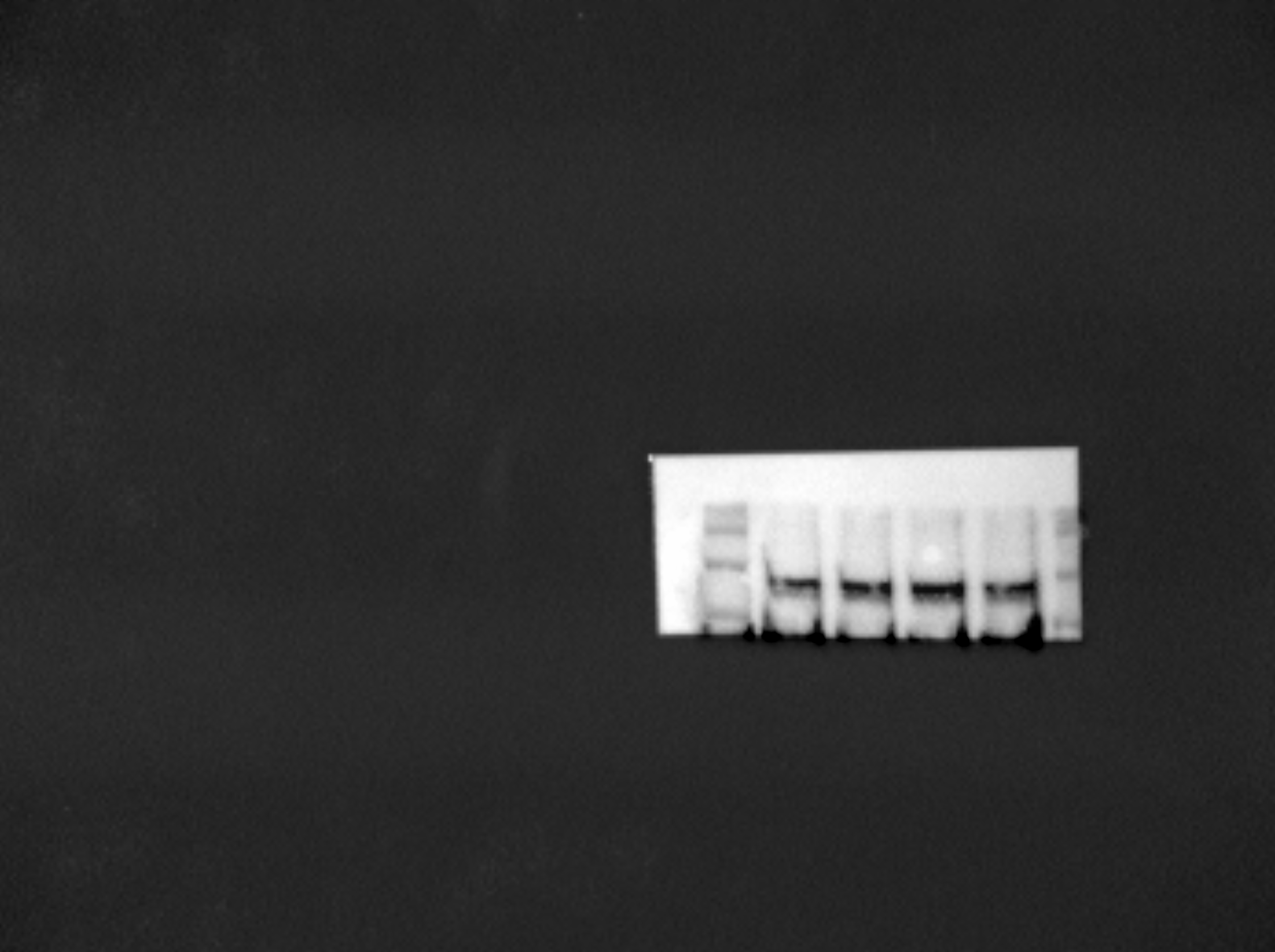

Supplement: Supplementary file 4 — Supplementary Material 4 [file 41598_2026_40491_MOESM4_ESM.tif]

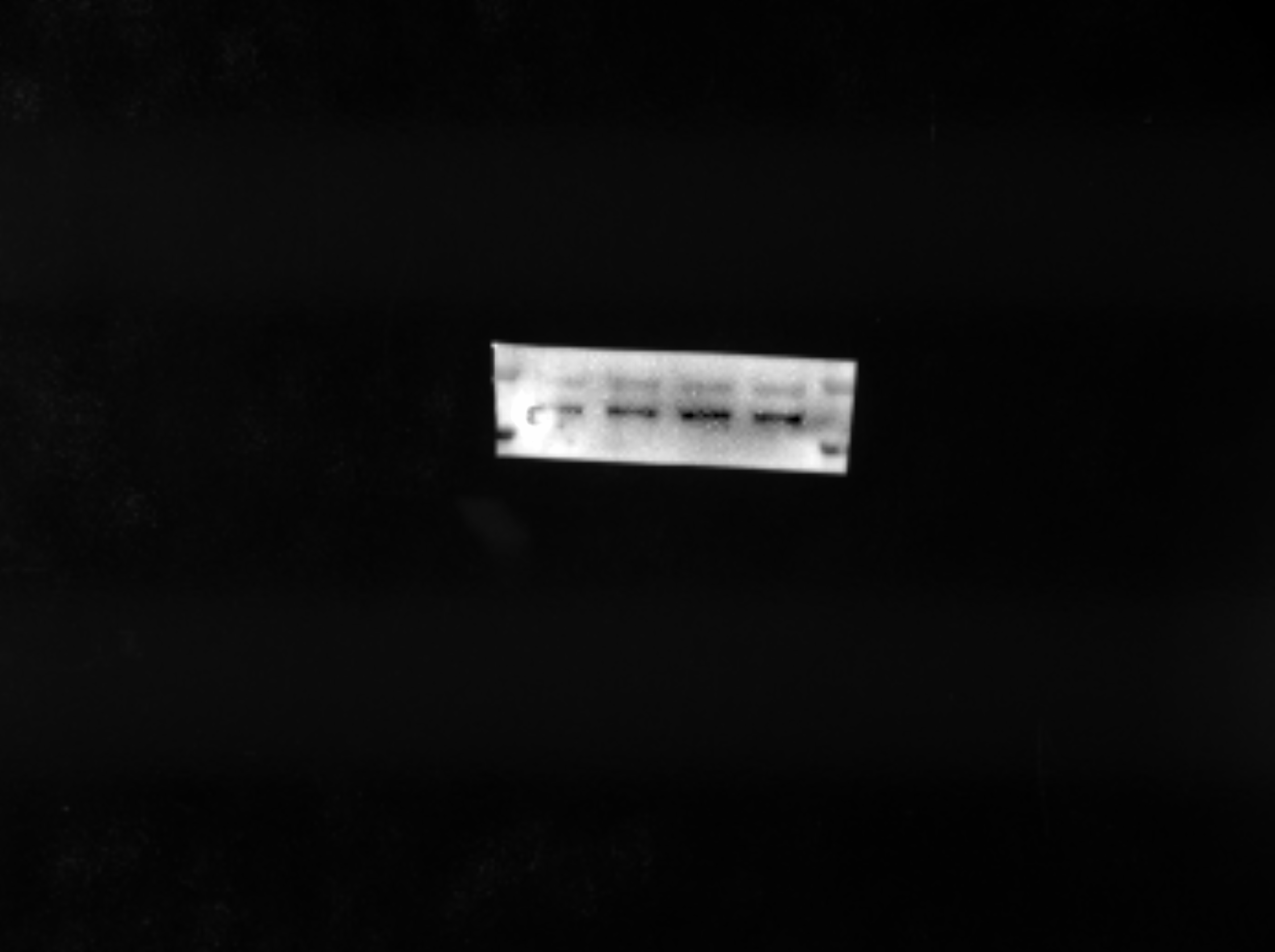

Supplement: Supplementary file 5 — Supplementary Material 5 [file 41598_2026_40491_MOESM5_ESM.tif]

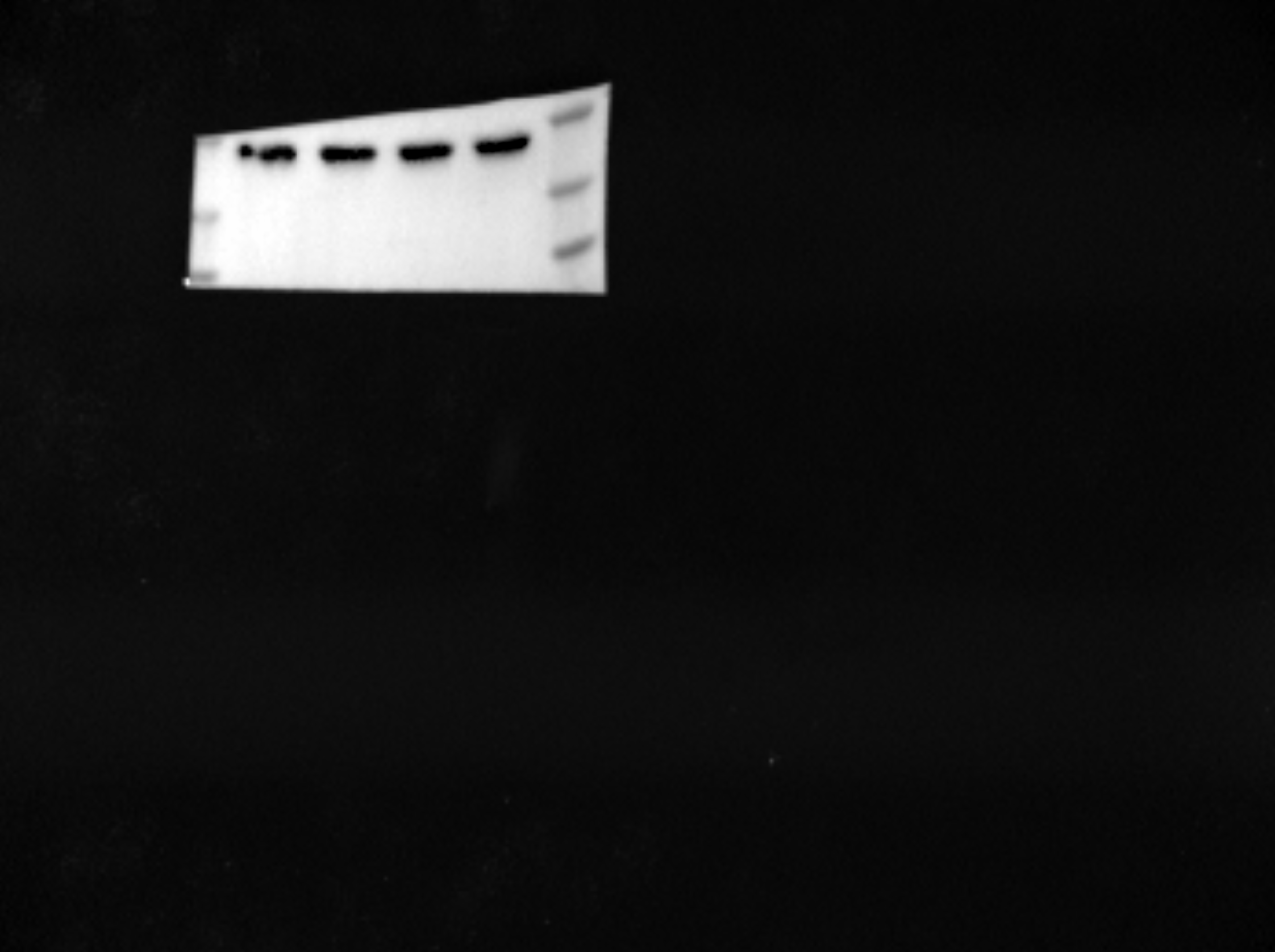

Supplement: Supplementary file 6 — Supplementary Material 6 [file 41598_2026_40491_MOESM6_ESM.tif]

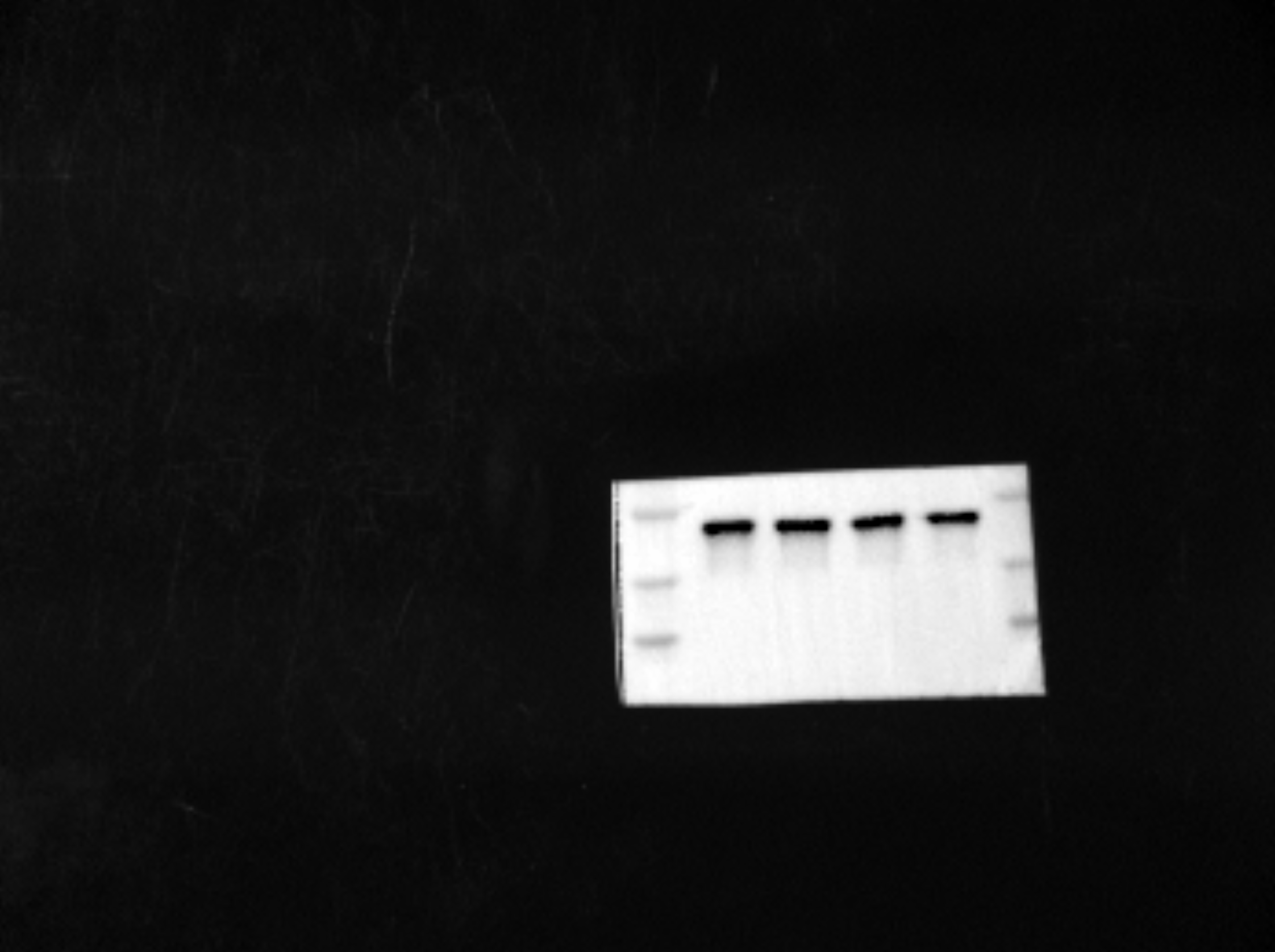

Supplement: Supplementary file 7 — Supplementary Material 7 [file 41598_2026_40491_MOESM7_ESM.tif]

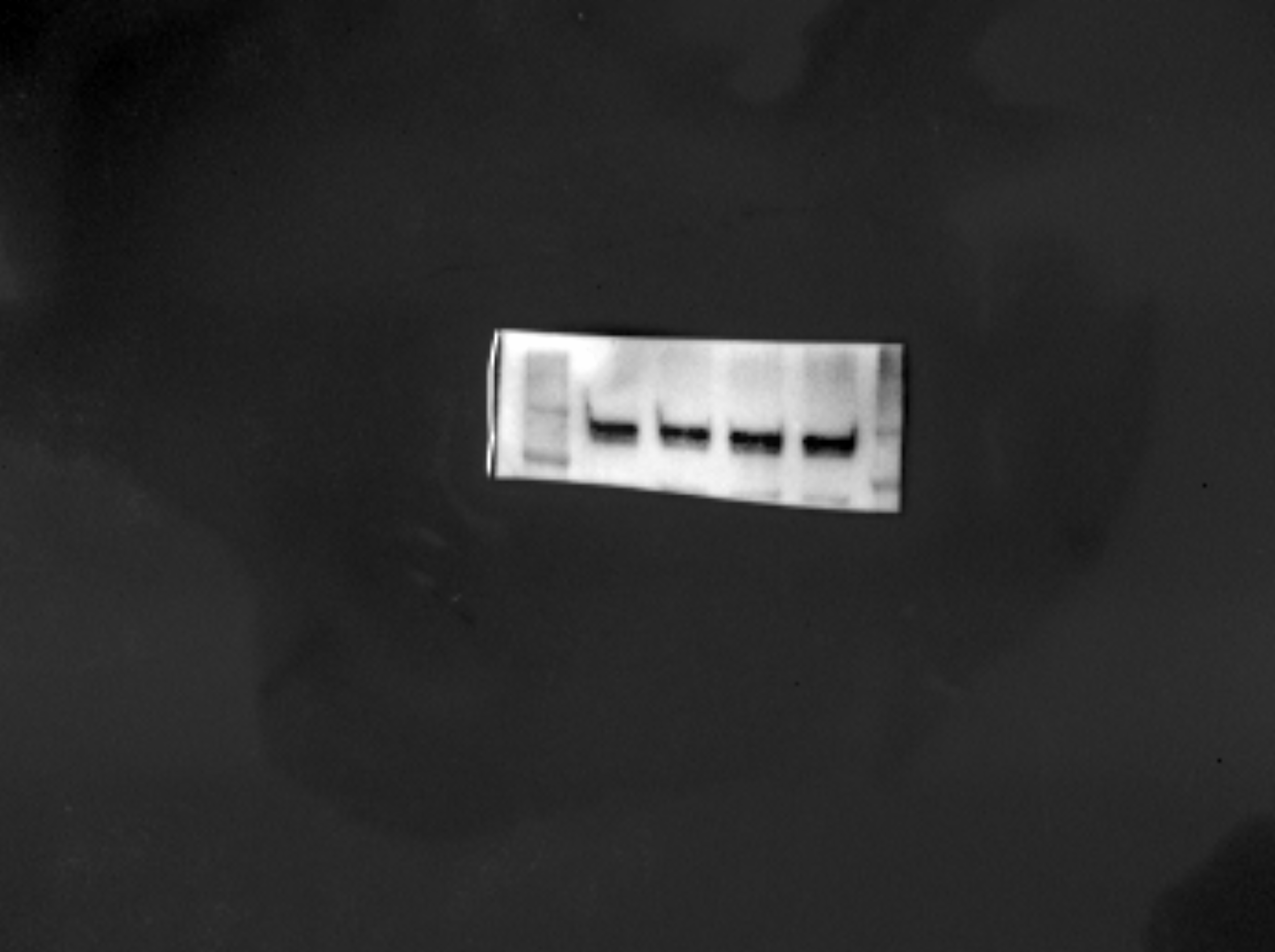

Supplement: Supplementary file 8 — Supplementary Material 8 [file 41598_2026_40491_MOESM8_ESM.tif]

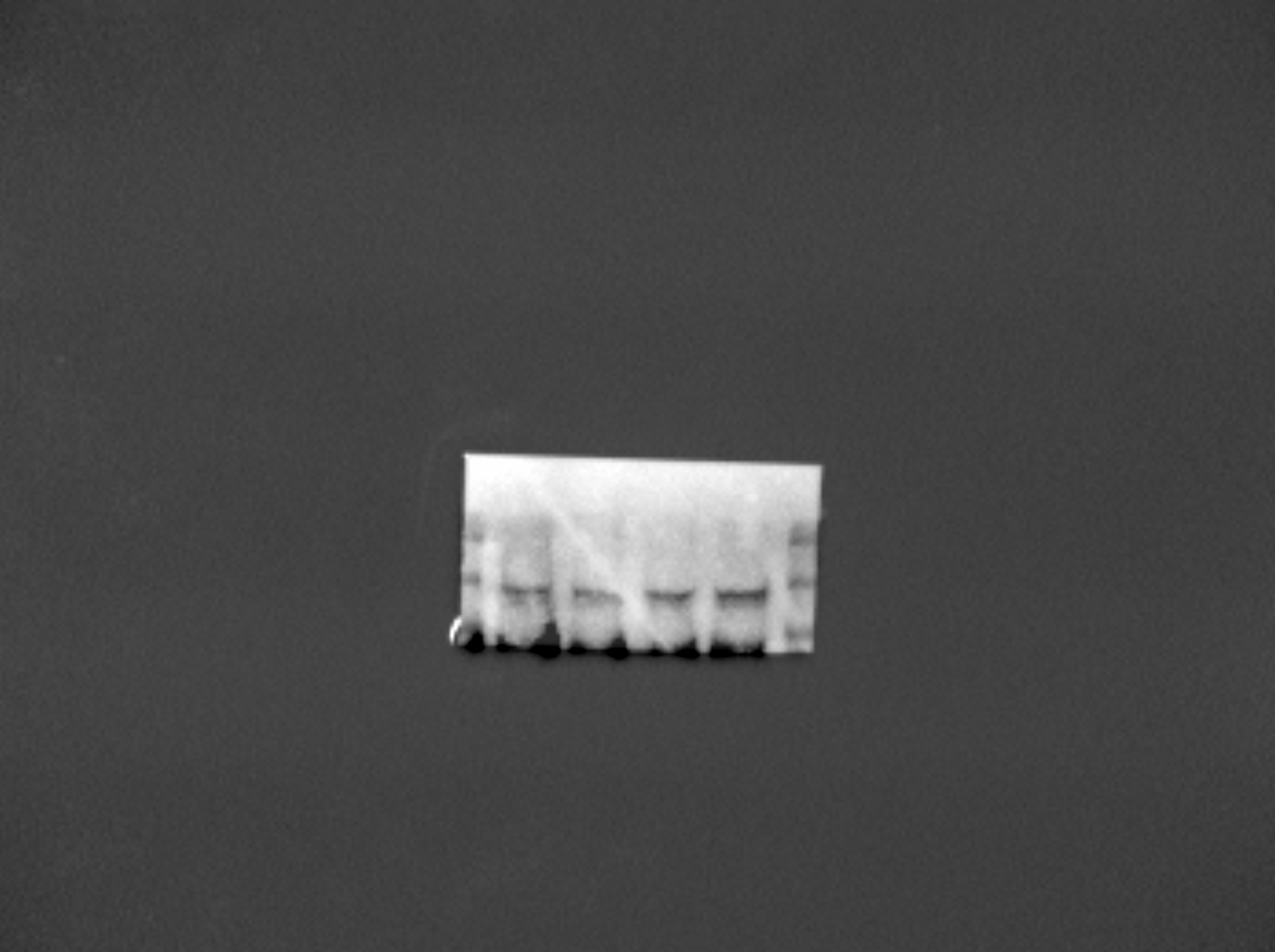

Supplement: Supplementary file 9 — Supplementary Material 9 [file 41598_2026_40491_MOESM9_ESM.tif]

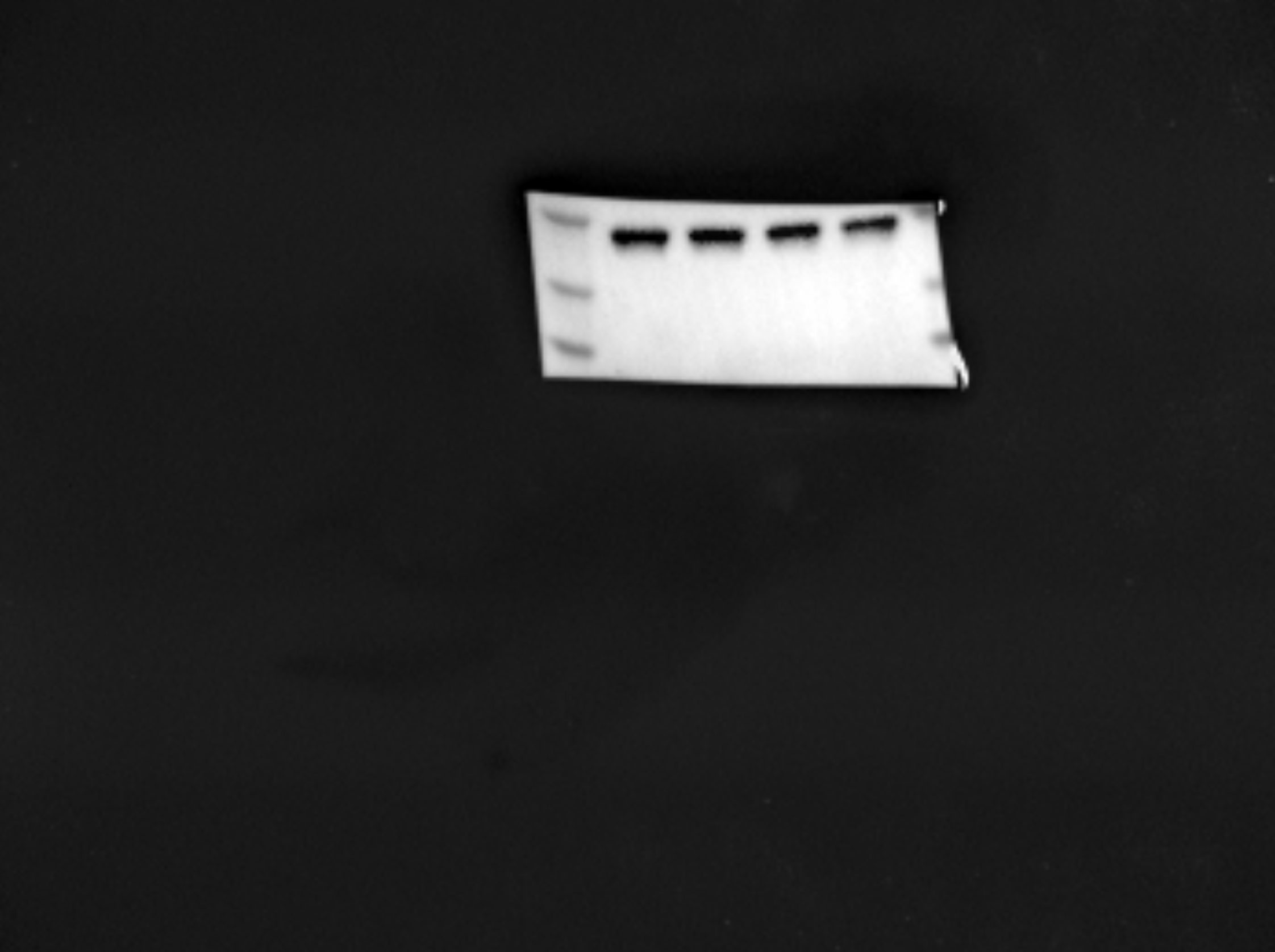

Supplement: Supplementary file 10 — Supplementary Material 10 [file 41598_2026_40491_MOESM10_ESM.tiff]

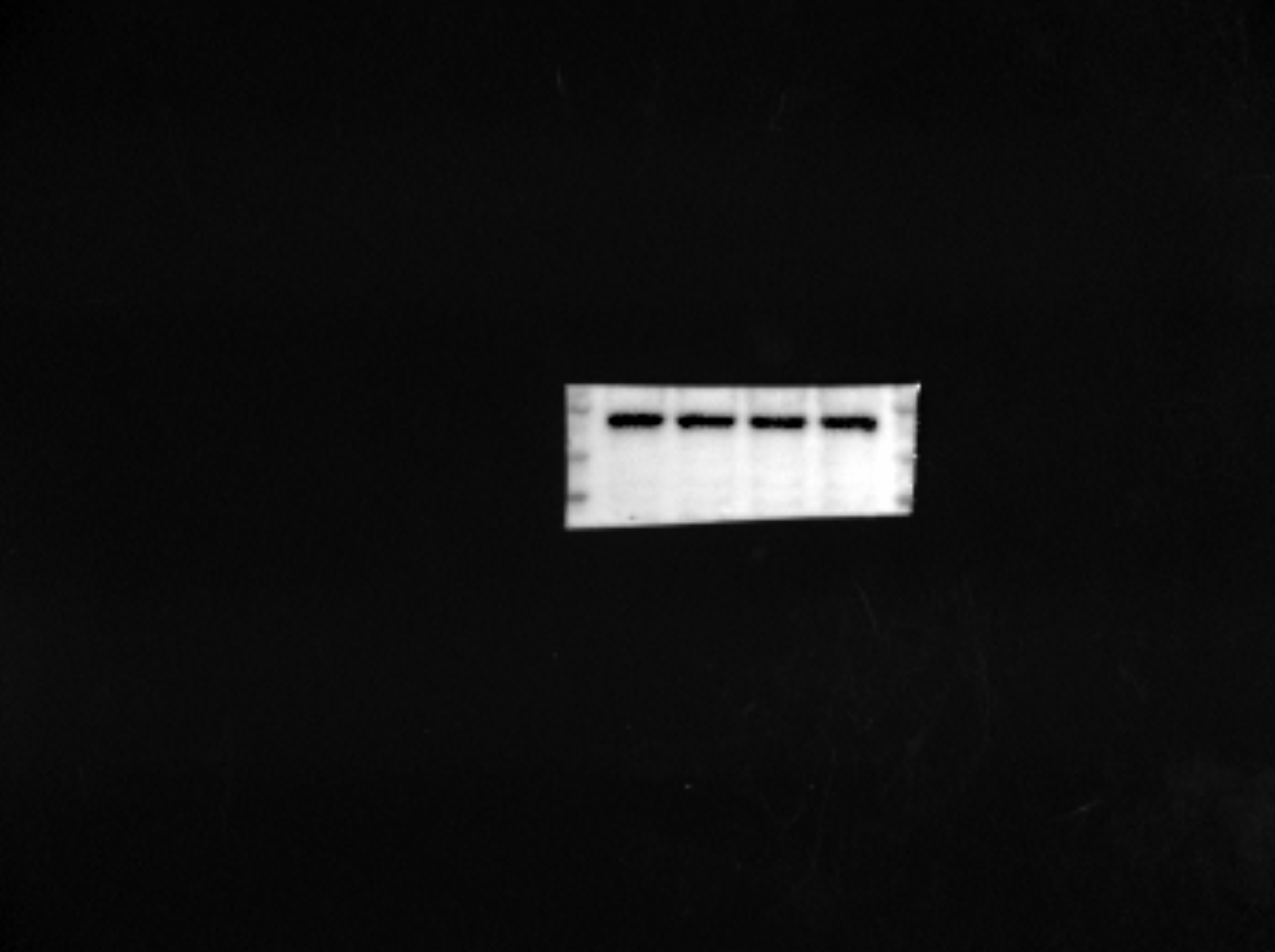

Supplement: Supplementary file 11 — Supplementary Material 11 [file 41598_2026_40491_MOESM11_ESM.tiff]

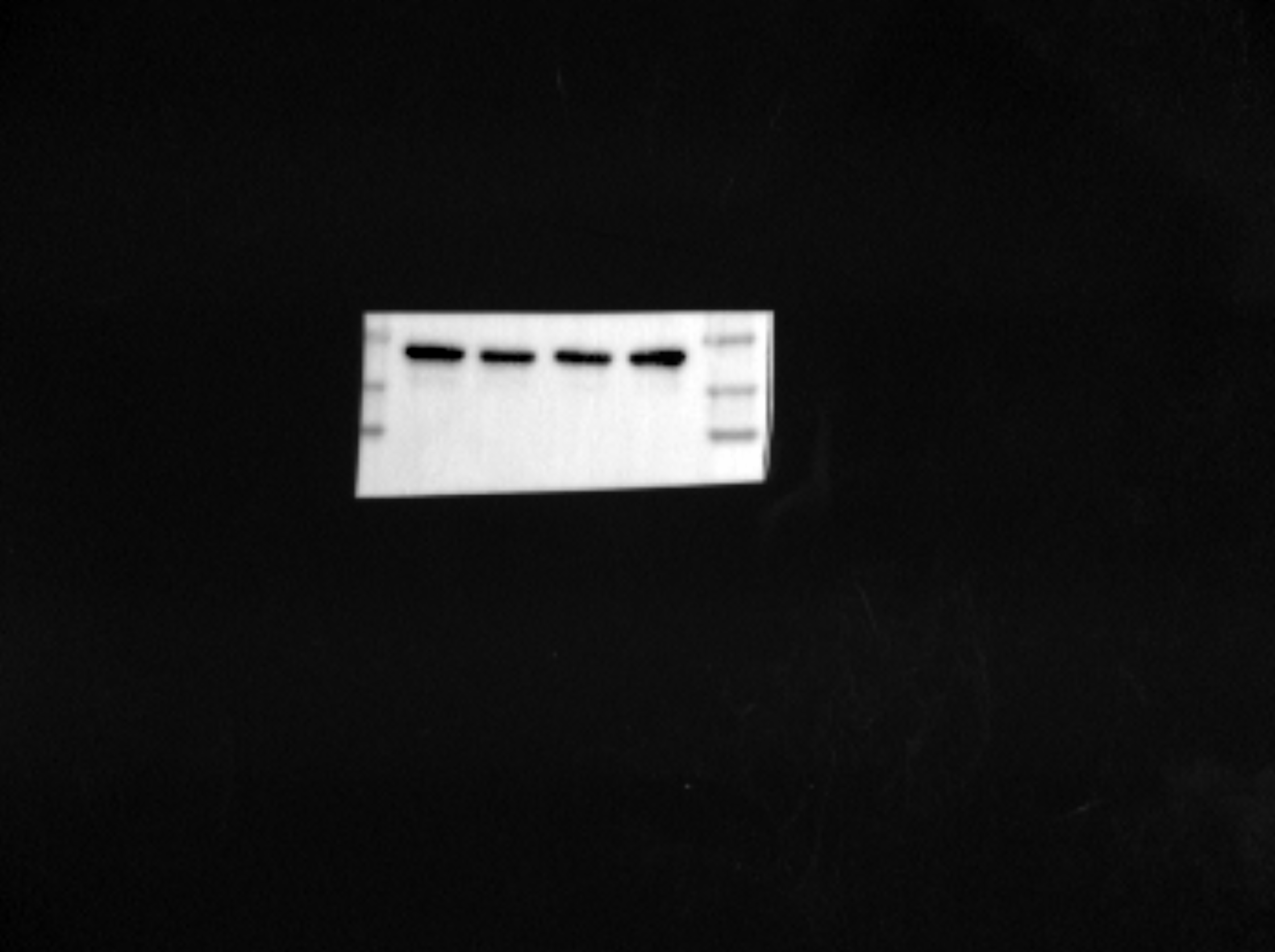

Supplement: Supplementary file 12 — Supplementary Material 12 [file 41598_2026_40491_MOESM12_ESM.tiff]

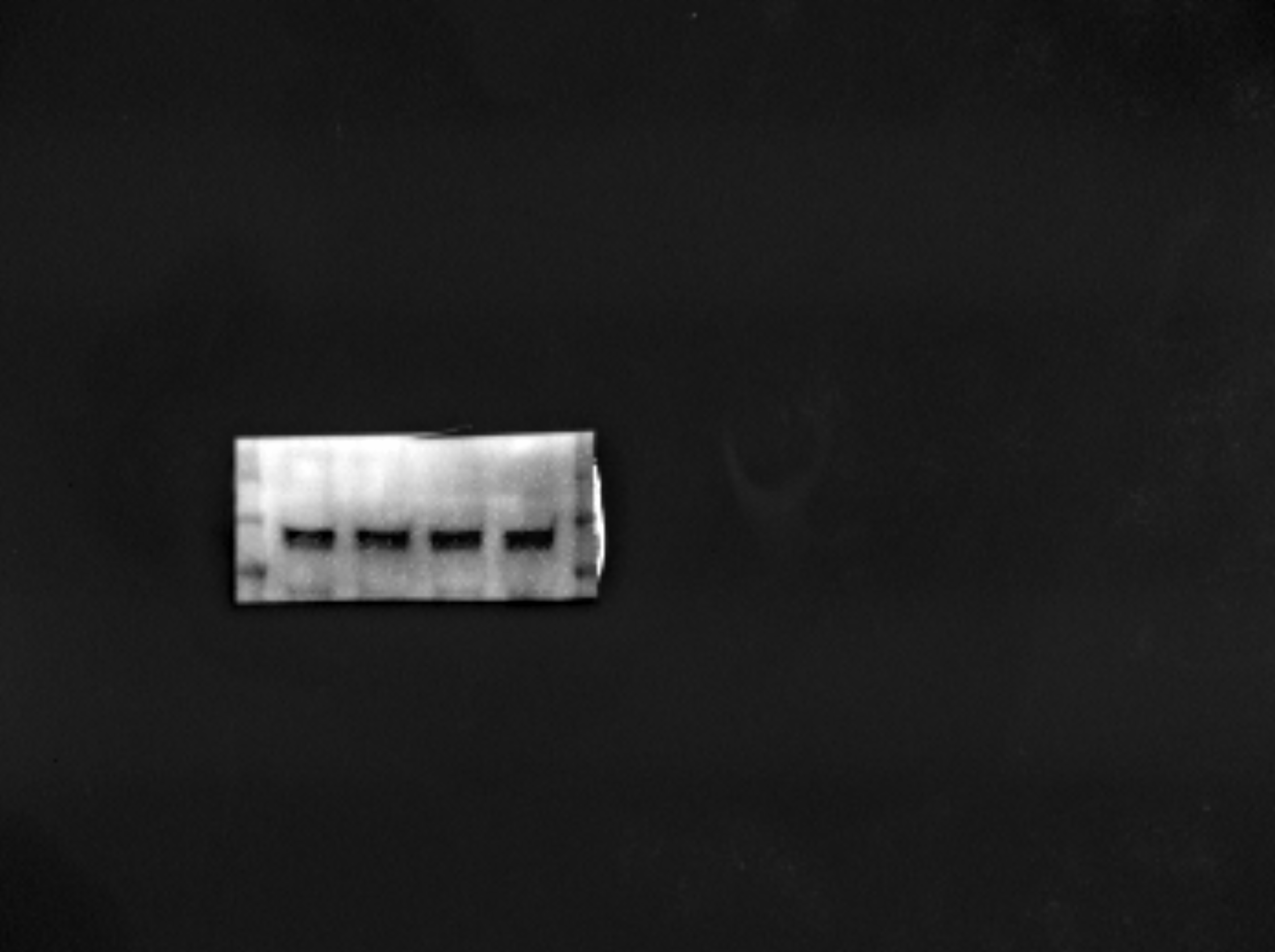

Supplement: Supplementary file 13 — Supplementary Material 13 [file 41598_2026_40491_MOESM13_ESM.tiff]

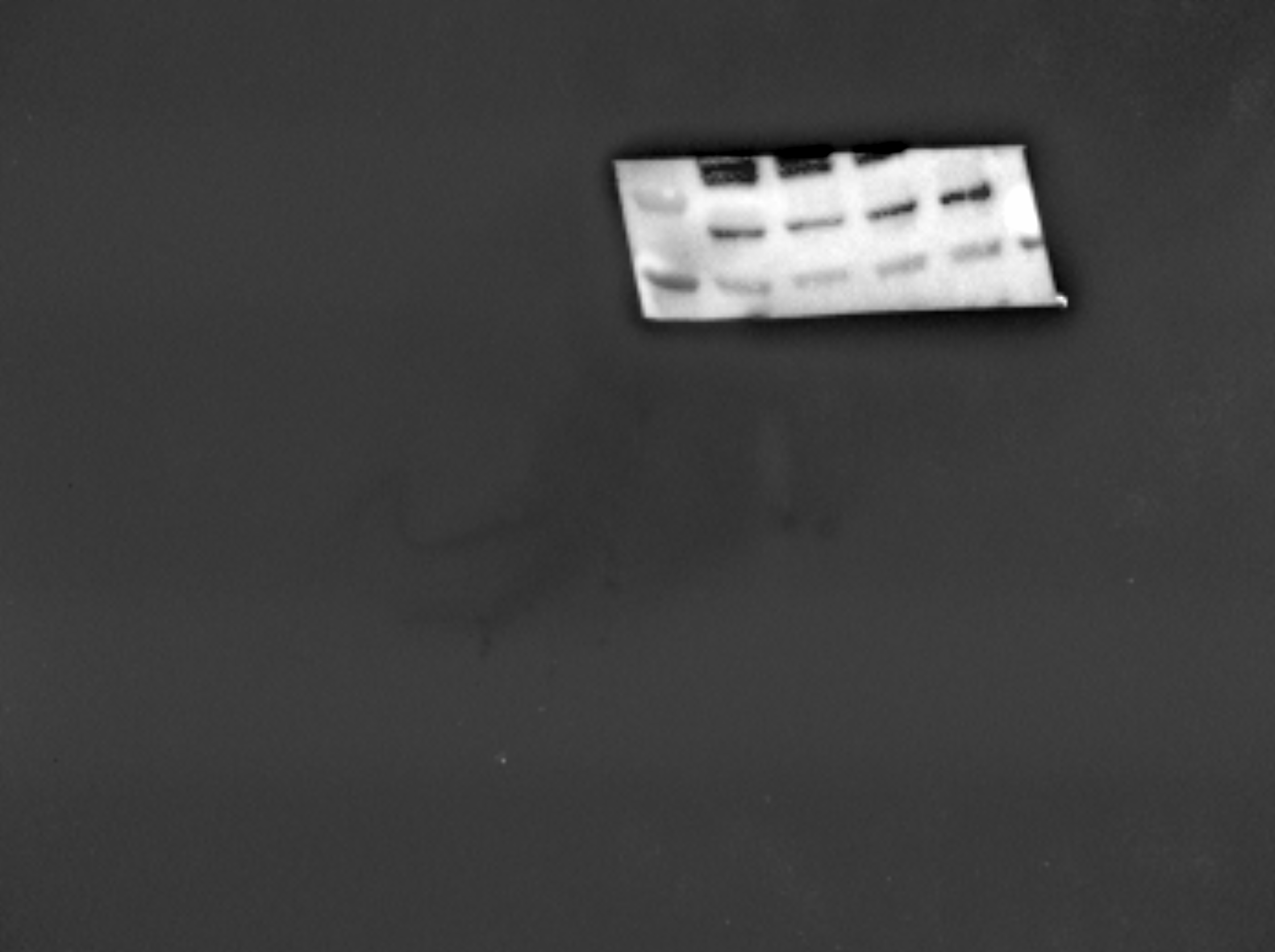

Supplement: Supplementary file 14 — Supplementary Material 14 [file 41598_2026_40491_MOESM14_ESM.tiff]

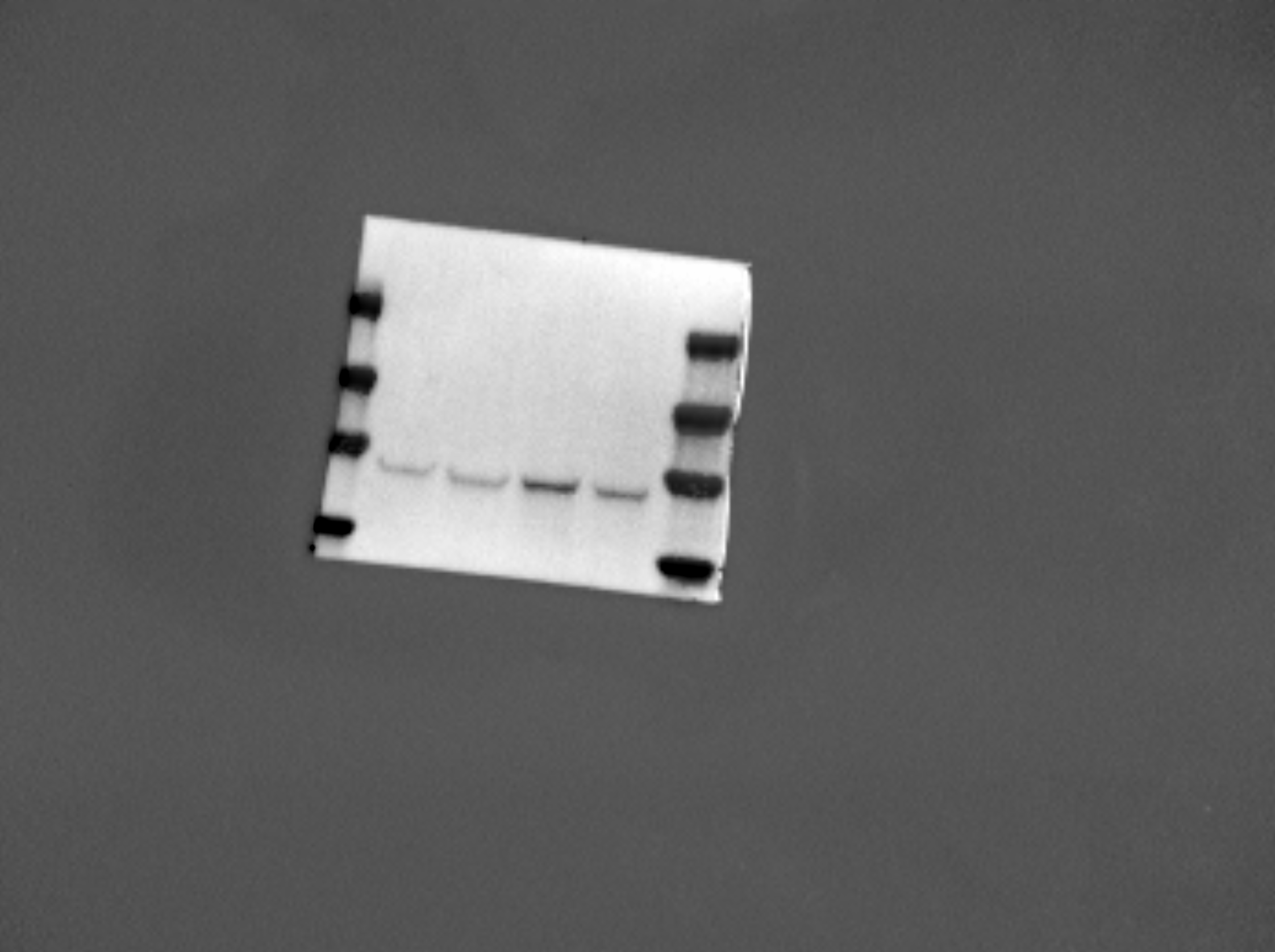

Supplement: Supplementary file 15 — Supplementary Material 15 [file 41598_2026_40491_MOESM15_ESM.tiff]

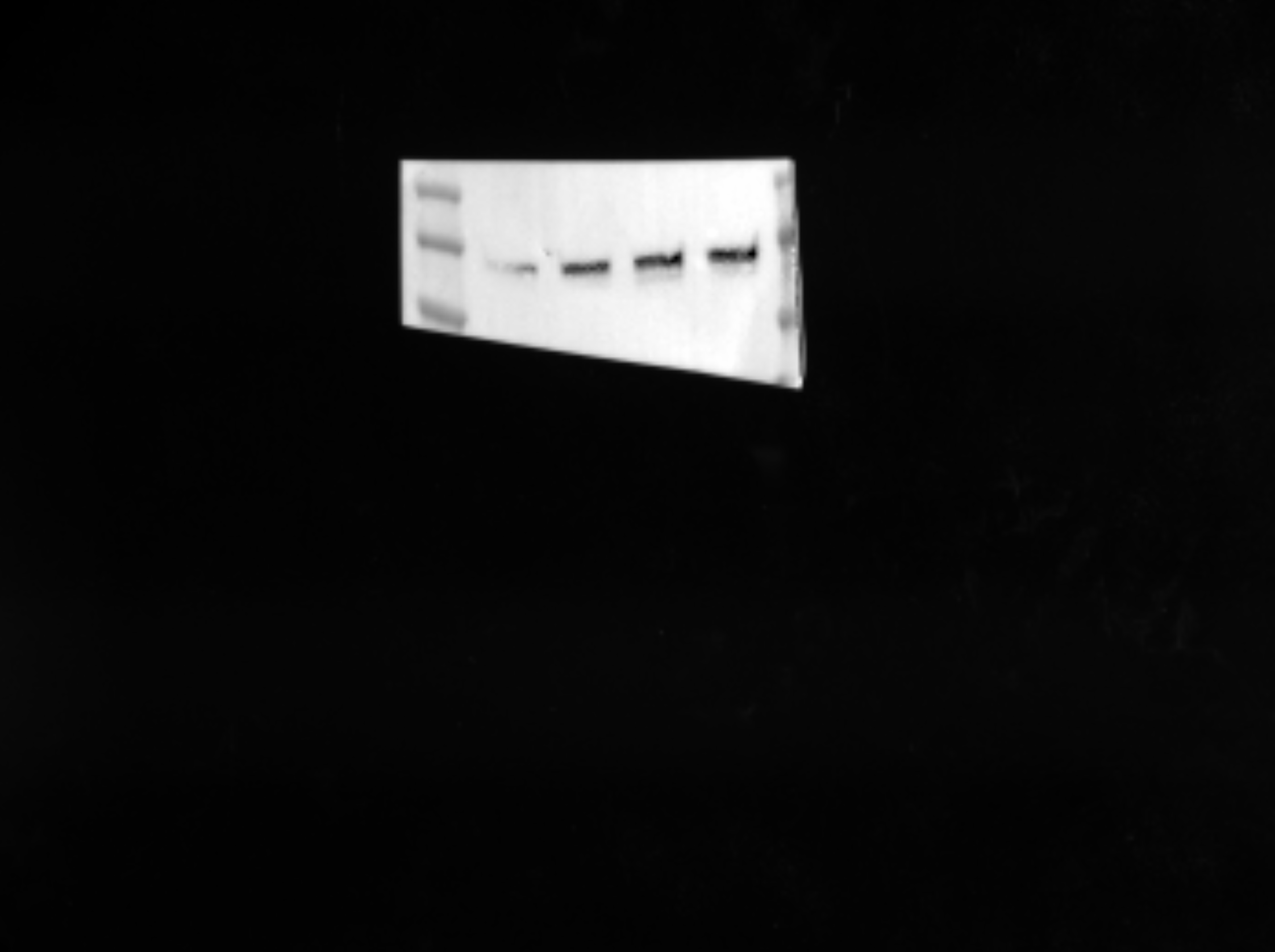

Supplement: Supplementary file 16 — Supplementary Material 16 [file 41598_2026_40491_MOESM16_ESM.tiff]

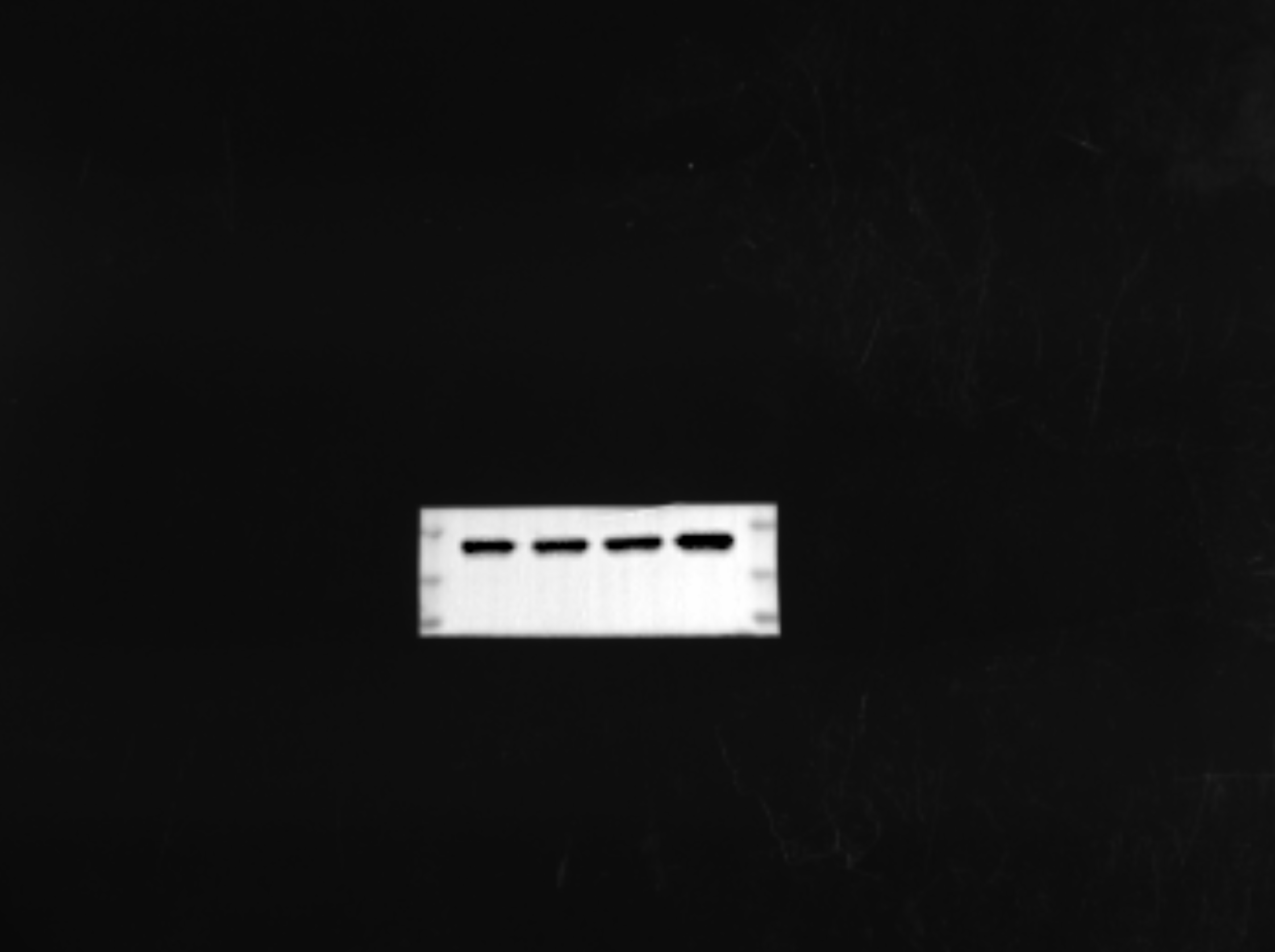

Supplement: Supplementary file 18 — Supplementary Material 18 [file 41598_2026_40491_MOESM18_ESM.tiff]

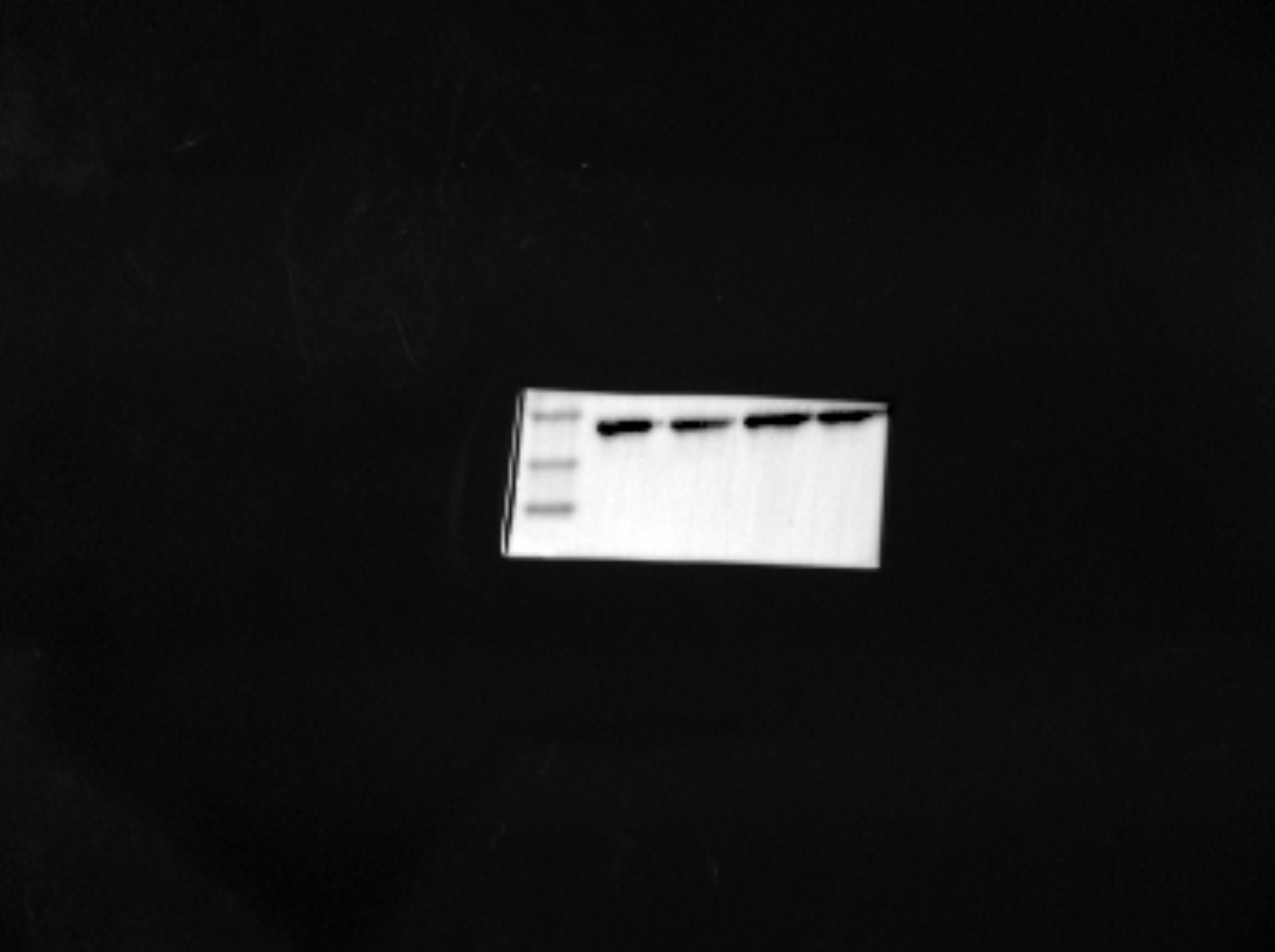

Supplement: Supplementary file 19 — Supplementary Material 19 [file 41598_2026_40491_MOESM19_ESM.tiff]

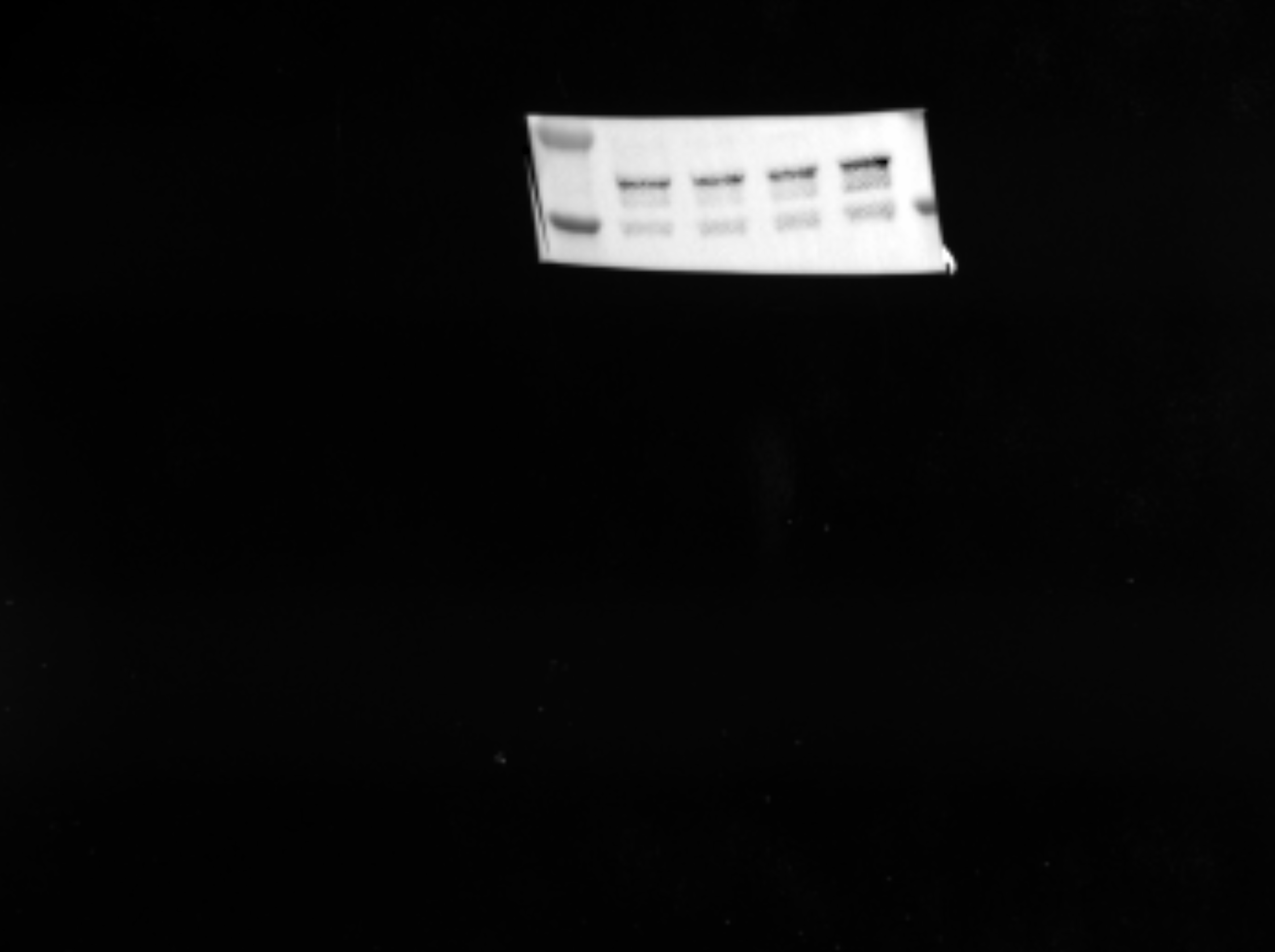

Supplement: Supplementary file 20 — Supplementary Material 20 [file 41598_2026_40491_MOESM20_ESM.tiff]

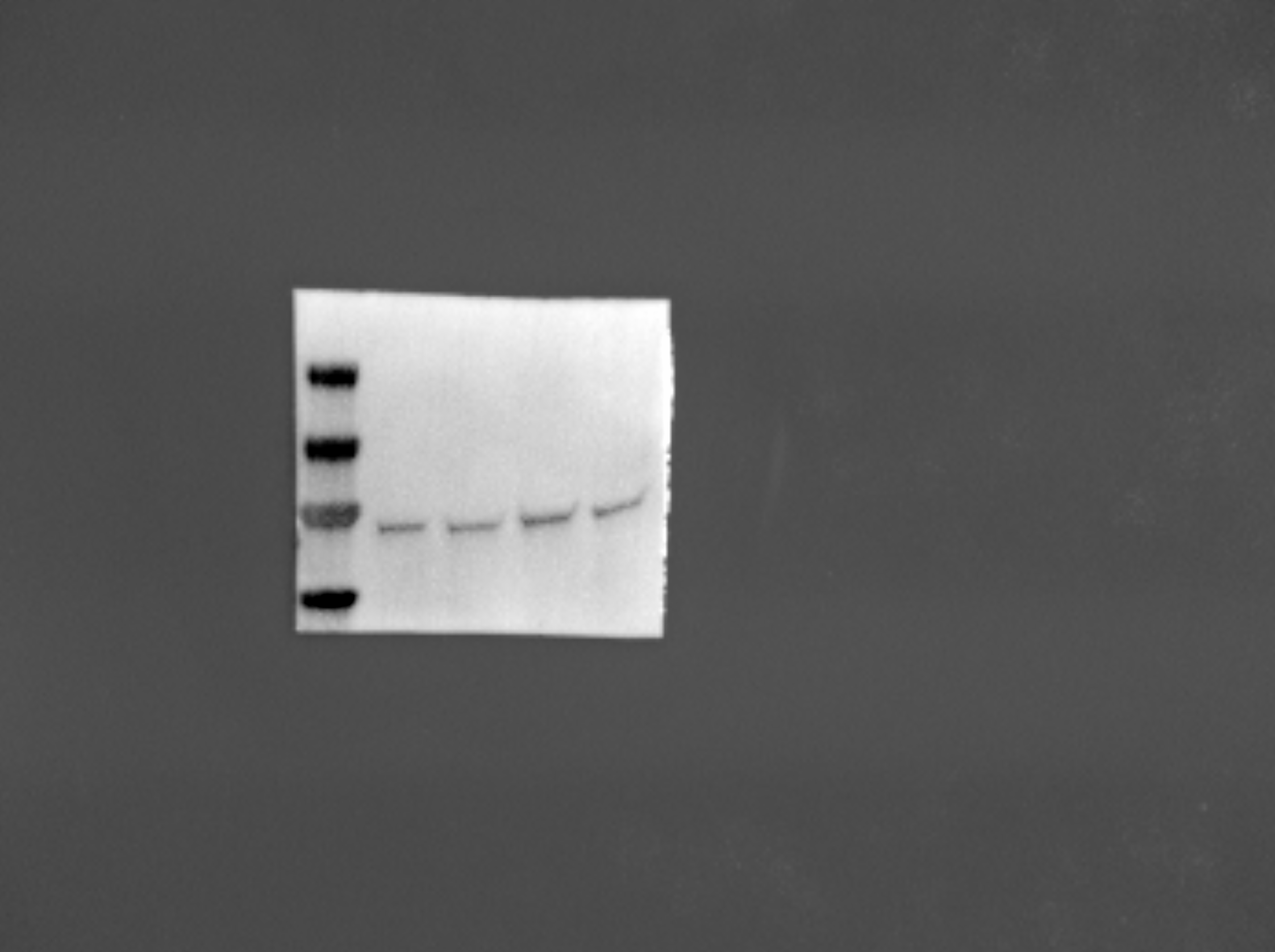

Supplement: Supplementary file 21 — Supplementary Material 21 [file 41598_2026_40491_MOESM21_ESM.tiff]

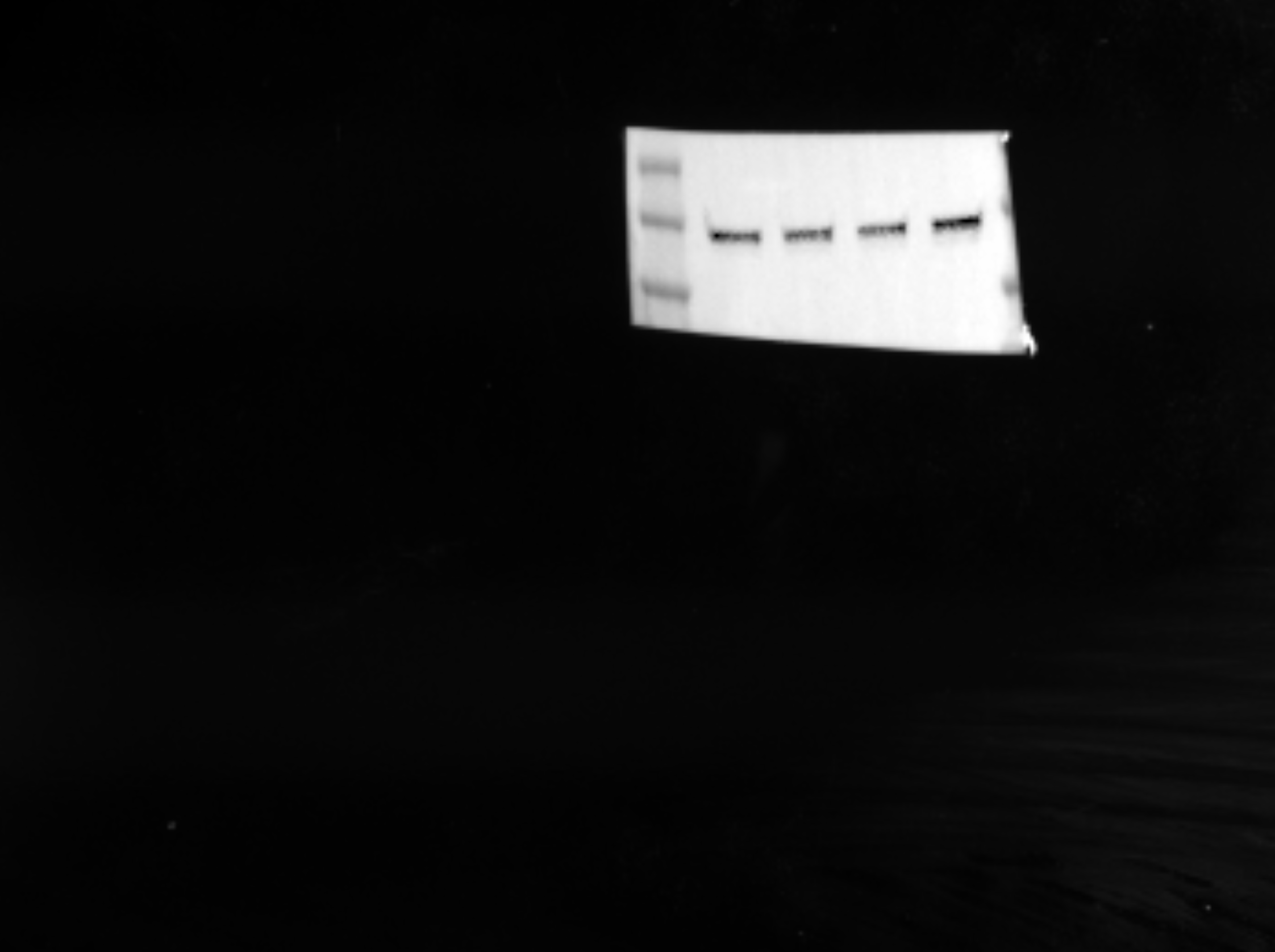

Supplement: Supplementary file 22 — Supplementary Material 22 [file 41598_2026_40491_MOESM22_ESM.tiff]
